# Transcriptome-based WGCNA reveals the molecular regulation of xylem plasticity in acclimation to drought and rewatering in mulberry

**DOI:** 10.3389/fpls.2024.1512645

**Published:** 2024-12-18

**Authors:** Yue Tian, Zeyang Zhai, Yujie Yang, Kaixin Zhang, Sang Ma, Jialing Cheng, Li Liu, Xu Cao

**Affiliations:** ^1^ Jiangsu Key Laboratory of Sericultural and Animal Biotechnology, School of Biotechnology, Jiangsu University of Science and Technology, Zhenjiang, China; ^2^ Key Laboratory of Silkworm and Mulberry Genetic Improvement, Ministry of Agriculture and Rural Affairs, Sericultural Scientific Research Center, Chinese Academy of Agricultural Sciences, Zhenjiang, China

**Keywords:** drought resistance, cambium, wood formation, vessel diameter, phytohormone, non-structural carbohydrates, *Morus*

## Abstract

Xylem plasticity is important for trees to coordinate hydraulic efficiency and safety under changing soil water availability. However, the physiological and transcriptional regulations of cambium on xylem plasticity are not well understood. In this study, mulberry saplings of drought-resistant Wubu and drought-susceptible Zhongshen1 were subjected to moderate or severe drought stresses for 21 days and subsequently rewatered for 12 days. The anatomical, physiological and transcriptional responses in wood and cambium were analyzed. Most parameters were not affected significantly under moderate drought for both cultivars. Severe drought led to decreased vessel lumen diameter and increased vessel frequency, increased starch and hemicellulose in wood of both cultivars. Notably, increased soluble sugars and lignin were detected only in wood of Wubu. In cambial zone, levels of starch, glucose, fructose, mannose and cytokinin were decreased in both cultivars, whereas soluble sugars were increased in Wubu but deceased in Zhongshen1. RNA-sequencing identified 1824 and 2471 differentially expressed genes in Wubu and Zhongshen1 under severe drought, respectively. These responses were partially recovered after rewatering. Weighted gene correlation network analysis identified modules of co-expressed genes correlated with the anatomical and physiological traits of wood and cambium, with the turquoise and green modules most strongly correlated with traits under drought or rewatering. These modules were enriched in gene ontology terms related to cell division, cytoskeleton organization, cell wall modification, dark respiration, vesicle transport and protein metabolism. Detailed gene expression patterns indicate that reprogramming of cambium activity was relatively similar in both cultivars, but at different scales. These findings provide important insights into the physiological and molecular mechanisms underlying xylem plasticity based on cambium and offer valuable references for breeding drought-resistant mulberry and other woody species in light of future drier climate scenarios.

## 1 Introduction

Drought is one of the most detrimental environmental factors affecting tree growth, productivity and even survival, as evidenced by increased forest mortality induced by intensified drought scenarios in recent decades (Allen et al., 2010; Xu et al., 2024). Xylem plasticity serves as an important mechanism in adapting water shortage for trees by maximizing water transport efficiency and reducing hydraulic risk in woody stems (Kooyers, 2015; Ahmad et al., 2018). The xylem of tree stem acts as the pathway for long-distance transport and allocation of water and minerals from roots to distal leaves (Meinzer et al., 2001). Xylem anatomy is not only closely coupled with tree hydraulic traits, but also affects wood properties important for its ecological and industrial utilizations in carbon sequestration, biofuels, and lumber and paper making (Li et al., 2021; Lu et al., 2024). Xylem is primarily composed of vessels specialized for water transport and fibers providing mechanical support for upright growth (Hacke et al., 2017). Vessel density and lumen diameter are the two most widely recognized anatomical traits, as described by the Hagen Poiseuille’s law demonstrating the fourth-power relationship between vessel diameter and hydraulic conductivity (Tyree and Zimmermann, 2002). However, vessels with wider lumen are more susceptible to drought-induced embolism which can leads to hydraulic failure and dieback of trees (Anderegg et al., 2012; Fang et al., 2021). Therefore, narrow vessels with comparable density are theoretically more advantageous under water deficit conditions in terms of balancing xylem hydraulic safety and efficiency (Sperry et al., 2006; Lübbe et al., 2022).

Xylem formation is closely related to vascular cambium activity under the regulation of a plethora of developmental and environmental signals (Wang et al., 2021). After the establishment of the vascular cambium and initiation of radial growth, cambium cells divide bifacially and subsequently differentiate into xylem cells inwards and phloem cells outwards (Růžička et al., 2015). Xylem formation involves four major steps: cell proliferation, cell expansion, cell wall deposition and programmed cell death (Plomion et al., 2001). The spatial arrangement and frequency of cell division within the cambial zone dictate the organization of cells within xylem tissue (Luo and Li, 2022). The ultimate size of vessels is related to the flexibility of the primary cell walls and the development of secondary cell wall (Zhu and Li, 2024). In recent years, the molecular dynamics driving wood formation from cambial zone has been gradually uncovered in model plants *Arabidopsis* and *Populus* (Wang et al., 2021). Multiple candidate regulators including phytohormones, peptides and transcriptional factors jointly control the formation, maintenance and proliferation of cambium (Zhu and Li, 2024). The regulatory network centered by CLAVATA3/EMBRYO SURROUNDING REGION-RELATED 41/44 (CLE41/44)-PXY-WUSCHEL-RELATED HOMEBOX 4 (WOX4) is the best-studied module regulating the maintenance of cambium cells (Hirakawa et al., 2010; Ji et al., 2009; Rodriguez-Zaccaro and Groover, 2019). Hybrid poplar (*Populus alba* × *P. glandulosa*) plants overexpressing *PagPXY* displayed more cambium cell layers and enhanced drought tolerance associated with increased capacity to alleviate reactive oxygen species compared to the wild type (Jiang et al., 2024). A recent study also identified *AT-HOOK MOTIF CONTAINING NUCLEAR LOCALIZED 15* (*AHL15*) in *Arabidopsis* operating in parallel to PXY-WOX4 pathway (Rahimi et al., 2022). Moreover, some other important regulators have been identified. The PaC3H17-PaMYB199 module in poplar can regulate cambium division by enhancing auxin level (Tang et al., 2020). Two MADS-box genes in poplar can modulate auxin homeostasis within cambial cells required by controlling *PIN-FORMED PROTEIN 5* (*PIN5*) expression (Zheng et al., 2021).

Until now, xylem plasticity has been documented in various forest tree species from the perspective of climate change (Rita et al., 2015). For instance, narrower or less vessels were observed after 2 years of experimental rainfall exclusion in lowland tropical trees (Tng et al., 2018). Similar patterns were also reported in three *Cedrela* species in drought years by analyzing tree-ring width and vessel traits (Rodríguez-Ramírez et al., 2022). The seasonal changes of cambium activity and wood formation have been reported (das Neves Brandes et al., 2015; Chao et al., 2022). However, investigations using combined anatomical, physiological and transcriptional methodologies to elucidate how cambium vitality participates in regulating wood anatomy in response to water deficit are quite scarce (Cassan-Wang et al., 2012; Perry et al., 2021; Zeng et al., 2023), but highly needed.

Mulberry is a fast-growing woody tree species whose leaves serve as the sole feed source for domesticated silkworm (Zhang et al., 2024). Mulberry trees have a wide range of geographical distribution benefiting from its robust adaptability to diversified environmental conditions (Rohela et al., 2020). In our previous studies, it was found that cultivar Wubu displayed better drought resistance at the physiological level to a short-term progressive soil dry-down than Zhongshen1 (Zhai et al., 2023), particularly to severe water deficit (Cao et al., 2020a). Anatomical analysis showed that this cultivar also has advantageously higher vessel frequency in petiole and stem wood conduit tissues than some other cultivars in the field (Cao et al., 2020b). Nevertheless, cultivar variability and xylem plasticity in response to different water availability remain to be investigated. In this work, we compared the general responses of both cultivars to different levels of drought stress and rewatering and targeted stem wood and cambium for anatomical, physiological and transcriptomic studies. By applying weighted gene co-expression network analysis (WGCNA), gene clusters or transcriptional regulators underpinning xylem plasticity were identified. Specifically, the following questions were addressed: (i) How do xylem anatomy and wood carbohydrates respond to drought and rewatering in mulberry? And (ii) Which genes or gene clusters are involved in regulating cambium activity to modulate xylem plasticity?

## 2 Materials and methods

### 2.1 Plant cultivation, drought treatment and harvests

One-year old cuttings (ca. 12 cm in length, 2 cm in diameter) of *Morus atropurpurea* cv. ‘Zhongshen1’ and *Morus alba* cv. ‘Wubu’ were induced to root (Du et al., 2016). Each rooted cutting was subsequently planted in a 10 L plastic pot that filled with commercial nutritional soil and perlite (1:1, v/v). The saplings were cultivated in a controlled-environment greenhouse with adequate irrigation for 6 months. For drought treatment, 36 uniform saplings of each cultivar were randomly allocated into three groups with 12 plants in each group. Plants in the three groups were exposed to 80% (well-watered, WW), 50% (moderate drought, MD) or 15% (severe drought, SD) of the saturated water content, respectively. To achieve the target soil water content of drought treatment, each pot was weighted every day and the amount of water equal to loss of transpiration and soil evaporation was added. The drought treatment lasted for 21 days. Subsequently, 6 plants were harvested in each group and the remaining plants were re-watered to 80% of the saturated soil water content. After 12 days, the remaining plants were also harvested noted as 80%r (WWr), 50%r (MDr), and 15%r (SDr). The pots were randomly rearranged every other day to reduce any disturbance of positions. The day/night temperature was 15/32°C, the relative air humidity was 60-80% and the maximum natural light intensity at the plant height was ca. 200 μmol m^-2^ s^-1^ during the experiment. For the harvest, each plant was separated into roots, bark, wood and leaves. Samples of stem cambial zone were collected by scraping the outer glutinous 1-2 mm layer of wood with razor blades (Teichmann et al., 2008). A bulk of the samples were weighed immediately and then oven-dried for 3 days at 72°C to determine biomass. The remaining samples were immediately frozen in liquid nitrogen and stored at −80°C until further analysis.

### 2.2 Gas exchange and growth performance

Leaf gas exchanges were recorded on the day before harvest on fully expanded leaves (LPI = 6-8) of six plants per group. Net photosynthetic rate (A), transpiration rate (E), stomatal conductance (g_s_), and intercellular carbon dioxide concentration (Ci) were measured between 8:00 AM and 10:00 AM using a CIRAS-3 Portable Photosynthesis System (PP Systems International Inc, Amesbury, MA, USA), equipped with a rectangle leaf chamber (18 mm × 25 mm). The following conditions were kept throughout the measurements: 1400 μmmol m^-2^ s^-1^ photo-synthetically active radiation, 400 ppm CO_2_ concentration, 200 μmol s^-1^ flow rates, 70% relative humidity and 30-36°C of leaf temperature. Instant water-use efficiency (WUE_i_) was evaluated as the ratio of A and E. The same leaves for gas exchange measurements were photographed and analyzed in Image J software (version 1.8.0; National Institutes of Health, Washington DC, USA) to obtain leaf area (LA). Shoot height and diameter (2 cm above shoot base), and leaf number of each plant were monitored every three days. The shoot height was recorded with a measuring tape and the ground diameter was measured with a digital slide caliper. The increments of shoot height, shoot diameter and leaf number were computed as the slope of the linear regression between days and these parameters. Root-shoot ratio was the ratio of the root biomass to aboveground biomass. Root length was defined as the longest single root measured by a ruler.

### 2.3 Analysis of wood anatomical characteristics

The wood anatomy was analyzed as previously reported (Cao et al., 2020b). 2-cm long stem segments were collected above the initial shoot height when the treatments begun. Half of the woody tissues were preserved in FAE (37% formalin: glacial acetic acid: 70% alcohol = 5:5:90, v:v:v) solution for freehand cross sectioning. The sections were mounted on glass slides and photographed under a light microscope (CX31, Olympus, Japan) equipped with a CCD (LY-WN-HP, Liyang Ltd., China). Hydraulically weighted vessel lumen diameter (VLD), vessel lumen area (VLA), vessel frequency (VF), xylem thickness (XT), pith radius (PR) and wood thickness (WT) were quantified for vessel properties. The proportions of pith, xylem and vessel areas were also computed. To estimate water transport capacity of the xylem vessels, predicted hydraulic conductivity per unit area (Kh) was calculated as Σr ^4^·mm^-2^, where r represented the radius of a vessel lumen (Tyree and Zimmermann, 2002). For wood maceration, wood chips were softened in Franklin solution (glacial acetic acid: 30% hydrogen peroxide = 1:1, v:v) at 65°C for 36 h. The macerated wood pieces were rinsed with de-ionized water and collapsed into single wood cells (vessel elements and fiber cells) by vigorous agitation with small glass balls in distilled water. The pulped wood solution was added to glass slides and stained with toluidine blue (0.05%, w/v). Images of wood cells were taken and vessel element length (VL) and fiber cell length (FL) were measured in Image J.

### 2.4 Determination of ROS, MDA and free proline in leaves

Reactive oxygen species (ROS) including superoxide anion (O_2_^•−^) and hydrogen peroxide (H_2_O_2_), and MDA in leaves were analyzed as described previously (Lei et al., 2007). Free proline was determined following the method of Liu et al. (2022).

### 2.5 Quantification of carbohydrates and phytohormones in wood and cambium

For wood and cambial samples, the concentrations of total soluble sugars and starch were determined spectrophotometrically as previously reported (Yemm and Willis, 1954). The concentrations of hemicellulose and lignin were quantified by content assay kits (D799082/022, Sangon Biotech, Shanghai, China). Furthermore, the concentrations of sucrose, glucose, fructose, mannose, maltose and galactose in cambial samples were analyzed on a high-performance liquid chromatography (HPLC) system (Rigol L300, Suzhou, China). Briefly, ca. 300 mg sample were extracted in 1 mL H_2_O overnight. The supernatants were separated with a NH_2_ column (4.6 mm × 250 mm, 5 μm particle size, Kromasil) to determine the concentrations of sucrose, glucose and fructose using a mobile phase of water. The other three monosaccharides including mannose, maltose, and galactose were analyzed using PMP derivatization method (Chen et al., 2023). Shortly, 50 μL of the supernatant were derivatized with the addition of 50 μL NaOH (0.6 M) and 100 μL PMP (0.5 M dissolved in methanol) for 100 min at 70°C. Then, 100 μL HCl (0.3 M) and 400 μL H_2_O was added with vigorous vortex. The reaction was extracted with 700 μL CHCl_3_ for three times and the upper aqueous phase was collected. The supernatant was separated with a reversed phase C_18_ column using a mobile phase of 20% acetonitrile and 80% phosphate buffer.

Indole-3-acetic acid (IAA), zeatin (ZA) and abscisic acid (ABA) in cambial samples were quantified using HPLC as described previously (Shang et al., 2019) with minor modifications (Chao et al., 2022). Briefly, ca. 100 mg samples were extracted in 1 mL of 80% pre-cooled methanol at 4°C in the dark overnight. The extraction was centrifuged and the supernatant was collected and adjusted to pH 2.8 using citric acid solution. The solution was mixed with ethyl acetate and the upper organic phase was collected and dried by N_2_. Afterwards, 0.5 mL MeOH was added to each sample and homogenized. The separation was performed by a mobile phase of 40% methanol in water and detected at 254 nm.

### 2.6 Library preparation and sequencing

Cambial zone samples from WW, SD, WWr and SDr were selected for transcriptomic analysis. Three biological replicates were produced by combing every two samples for each group. Total RNAs were isolated using RNA Plus Reagent (Takara, Japan) and the integrity was verified using an Agilent 2100 BioAnalyzer (Agilent, Santa Clara, CA, USA). mRNA was enriched, fragmented and reverse transcribed into cDNA with the addition of a single ‘A’ base and ligation of a bubble adapter. The products were purified and enriched with PCR amplification. The PCR products were dissociated into single strand and convert to a single strand circle DNA (ssCirc) using splint oligo primers, which generated the final library. DNA nanoballs (DNBs) were generated with the ssCirc by rolling circle replication to enlarge the fluorescent signals at the sequencing process. The DNBs were loaded into the patterned nanoarrays and single-end read of 50 bp were read through on the BGISEQ-500 sequencing platform (BGI, Shenzhen, China).

### 2.7 Bioinformatics and data analysis

The SOAPnuke software (v1.5.2, BGI) was utilized to filter out adapter sequences, reads with over 10% unknown nucleotides and low-quality reads with over 50% low SQ value (≤15) bases (Cock et al., 2010). Data analysis was performed according to the procedures in the previous publications (Luo et al., 2020; Li et al., 2024b) with minor modifications. The clean data were aligned to the published *Morus atropurpurea* cv. ‘Zhongshen1’ reference genome (Xia et al., 2024) using the Subread package in R (Liao et al., 2019). The gene abundance of each sample was quantified using featureCounts function in Subread package. For each transcription region, an FPKM (fragment per kilobase of transcript per million mapped reads) value was calculated using fpkm function in edgeR (Robinson et al., 2010). Principal component analysis (PCA) and correlation analysis were performed in R to reveal associations among treatments. Differential expression analysis was performed by DESeq2 software between two different groups (Love et al., 2014). Differentially expressed genes (DEGs) were identified based on an absolute fold change of ≥2 and a false discovery rate (FDR) less than 0.05. The FDR was set by adjusting the p-values using Benjamini and Hochberg’s method. The DEGs were annotated using protein sequences as queries against *Arabidopsis* protein sequence dataset (http://www.arabidopsis.org/) by local BLASTp as suggested (Luo et al., 2016). The enrichment analyses of Gene Ontology (GO) and Kyoto Encyclopedia of Genes and Genomes (KEGG) were performed with clusterprofiler packages in R (Wu et al., 2021; Luo et al., 2024). Gene co-expression analysis was conducted with WGCNA packages in R following Langfelder and Horvath (2008) with minor modifications (Luo et al., 2021).

### 2.8 DEGs validation using qRT-PCR

Fifteen DEGs were randomly selected to validate the RNA-Seq analysis data, and the gene specific primers are presented in **Supplementary Table S1**. For each treatment, RNA samples of three biological replicates for RNA-seq were used for quantitative real time PCR (qRT-PCR) validation. The first-strand cDNA was synthesized using a PrimeScript™ RT reagent kit with gDNA Eraser (RR047A, Takara, Japan). qRT-PCR was performed on an ABI StepOnePlus™ Real-Time PCR System (Applied Biosystems, Massachusetts, USA) using TB Green™ Premix Ex Taq II (RR820A, TaKaRa, Japan). Actin was used as the internal control gene. Three technical replicates were used for each sample. The relative expression levels were calculated by the 2^−ΔΔCt^ method (Livak and Schmittgen, 2001).

### 2.9 Statistical analysis

Statistical tests were performed in SPSS statistics 22 (IBM, NY, USA). All the variables were checked for normality before statistical analysis. To examine the effects of cultivar, water supply and their interactions on the variables, two-way ANONAs were used with cultivar (C) and water regime (W) as two main factors. Differences between means were considered significant when the *P*-value was less than 0.05. Six biological replicates were used for morphophysiological and anatomical analysis.

## 3 Results

### 3.1 Gas exchange, growth performance and overall drought responses

Under moderate drought, no obvious changes were found in gas exchange and growth parameters except for a significant decrease of E in Wubu. Zhongshen1 had significantly reduced E and g_s_, and increased WUE under MD (**Table 1**). SD dramatically effected the gas exchange and growth of both cultivars (**Table 1**). The increments of shoot height, shoot diameter and leaf number of Wubu decreased by 44.77%, 76.92% and 46.58%, respectively. This growth inhibition was more pronounced in Zhongshen1, reaching up to 60.89%, 83.33% and 67.86%, respectively. Gas exchange parameters and growth performance were not fully restored after rehydration for severely stressed saplings of both cultivars. Only the growth of shoot height and new leaf emergence recovered back to the similar levels as control.

**Table 1:** Effects of drought and re-watering on gas exchange and growth of drought-resistant Wubu and drought-susceptible Zhongshen1 exposed to 80, 50 and 15% field capacity for 21 days and re-watered to 80% field capacity (denoted as 80%r, 50%r and 15%r) for 12 days, respectively.

Variables	Wubu			Zhongshen1			ANOVA		
	80%	50%	15%	80%	50%	15%	C	W	C × W
Net photosynthetic rate (μmol m^-2^s^-1^)	23.37 ± 0.24 a	22.73 ± 0.43 ab	10.18 ± 0.38 d	20.65 ± 0.53 c	21.60 ± 0.46 bc	10.65 ± 0.51 d	**	****	**
Intercellular CO_2_ contents (μmol mol^-1^)	326.33 ± 1.98 a	321.83 ± 2.95 a	234.00 ± 21.89 b	329.00 ± 1.21 a	319.83 ± 1.74 a	208.00 ± 8.44 c	ns	****	ns
Transpiration rate (μmol m^-2^s^-1^)	8.17 ± 0.02 a	7.51 ± 0.22 b	3.05 ± 0.08 c	8.32 ± 0.05 a	7.15 ± 0.19 b	2.70 ± 0.36 c	ns	****	ns
Stomatal conductance (μmol m^-2^s^-1^)	1002.50 ± 36.41 bc	1064.67 ± 5.14 b	95.50 ± 2.25 d	1205.83 ± 55.29 a	953.83 ± 36.92 c	94.50 ± 7.03 d	ns	****	****
Water use efficiency	2.87 ± 0.03 b	3.03 ± 0.08 b	3.60 ± 0.17 a	2.43 ± 0.07 c	3.02 ± 0.08 b	3.53 ± 0.11 a	*	****	*
Height increment (cm day^-1^)	2.39 ± 0.03 a	2.04 ± 0.05 a	1.32 ± 0.15 c	2.25 ± 0.01 ab	2.39 ± 0.06 a	0.88 ± 0.12 d	ns	****	****
Shoot diameter increment (mm day^-1^)	0.13 ± 0.00 b	0.12 ± 0.01 b	0.03 ± 0.01 c	0.18 ± 0.01 a	0.17 ± 0.01 a	0.03 ± 0.01 c	****	****	**
Leaf number increment(day^-1^)	0.73 ± 0.06 a	0.64 ± 0.02 ab	0.39 ± 0.06 c	0.56 ± 0.03 b	0.54 ± 0.02 b	0.18 ± 0.03 d	****	****	ns
	80%r	50%r	15%r	80%r	50%r	15%r			
Net photosynthetic rate (μmol m^-2^s^-1^)	20.15 ± 0.47 a	19.30 ± 0.34 a	17.40 ± 0.67 b	17.58 ± 0.37 b	17.18 ± 0.54 b	16.98 ± 0.54 b	****	**	ns
Intercellular CO_2_ contents (μmol mol^-1^)	270.33 ± 2.86 b	247.67 ± 6.86 c	210.17 ± 8.48 d	302.33 ± 4.36 a	272.00 ± 8.00 b	211.20 ± 10.42 d	**	****	ns
Transpiration rate (μmol m^-2^s^-1^)	3.44 ± 0.05 a	3.13 ± 0.12 ab	2.27 ± 0.05 c	3.27 ± 0.13 ab	3.18 ± 0.12 ab	2.27 ± 0.05 c	ns	****	ns
Stomatal conductance (μmol m^-2^s^-1^)	305.67 ± 11.48 b	228.83 ± 16.04 c	130.67 ± 6.56 d	351.33 ± 21.87 a	239.5 ± 18.18 c	131.80 ± 9.44 d	ns	****	ns
Water use efficiency	5.87 ± 0.13 b	6.20 ± 0.28 b	7.53 ± 0.29 a	5.40 ± 0.13 b	5.47 ± 0.39 b	7.48 ± 0.28 a	ns	****	ns
Height increment (cm day^-1^)	1.96 ± 0.08 ab	1.86 ± 0.06 ab	1.8 ± 0.07 b	1.94 ± 0.08 ab	2.15 ± 0.04 a	1.74 ± 0.16 b	ns	*	ns
Shoot diameter increment (mm day^-1^)	0.14 ± 0.01 b	0.12 ± 0.01 b	0.08 ± 0.01 c	0.17 ± 0.01 a	0.17 ± 0.01 a	0.10 ± 0.01 c	***	****	ns
Leaf number increment(day^-1^)	0.66 ± 0.03 a	0.58 ± 0.02 ab	0.56 ± 0.06 ab	0.42 ± 0.02 c	0.44 ± 0.02 c	0.39 ± 0.03 c	****	ns	ns

The bar indicates mean ± SE. Different letters after the mean values in the same row indicate significant difference. ANOVAS of cultivars (C), soil water content (W) and their interaction (C × W) are also indicated. *P < 0.05; **P < 0.01; ***P < 0.001; ****P < 0.0001; ns, not significant. 80%, 50%, 15% indicate control, moderate drought and severe drought treatment. 80%r, 50%r, 15%r indicate the corresponding re-watering treatment.

Slight but non-significant variations were found in biomass or morphology of both cultivars under MD (**Table 2**). Under SD, both varieties displayed significant decline in aboveground biomass, leaf area and root length, while Zhongshen1 had higher growth inhibition compared to Wubu (**Table 2**). The stem and wood biomass of Zhongshen1 decreased by 61.92% and 67.81% respectively, while those of Wubu only decreased by 44.39% and 52.47%. SD also significantly increased the root-shoot ratio of both cultivars. Regardless of the water regimes, Wubu always had larger non-leafy biomass and root length, and smaller leaf area, suggesting its distinct biomass allocation preference to sink organs. Most of the drought induced growth inhibition was not reversed after rewatering in both mulberry cultivars.

**Table 2:** Effects of drought and re-watering on morphology parameters and biomass of drought-resistant Wubu and drought-susceptible Zhongshen1 exposed to 80, 50 and 15% field capacity for 21 days and re-watered to 80% field capacity (denoted as 80%r, 50%r and 15%r) for 12 days, respectively.

Variables	Wubu			Zhongshen1			ANOVA		
	80%	50%	15%	80%	50%	15%	C	W	C × W
Leaf biomass(g)	27.55 ± 1.09 b	25.57 ± 1.95 b	16.84 ± 1.17 c	32.95 ± 1.81 a	28.83 ± 1.10 ab	16.05 ± 1.31 c	*	****	ns
Stem biomass (g)	25.48 ± 1.49 a	23.37 ± 2.41 a	14.17 ± 0.86 bc	23.45 ± 2.01 a	19.79 ± 1.31 ab	8.93 ± 0.64 c	**	*	ns
Wood biomass (g)	16.58 ± 1.16 a	14.25 ± 1.79 ab	7.88 ± 0.74 c	13.73 ± 1.45 ab	11.49 ± 1.23 b	4.42 ± 0.35 c	**	****	ns
Root biomass (g)	14.21 ± 0.86 a	14.04 ± 0.67 a	12.94 ± 0.81 ab	10.81 ± 0.75 bc	10.57 ± 0.13 c	8.64 ± 0.35 c	***	****	****
Aboveground biomass (g)	53.03 ± 2.30 a	48.94 ± 4.31 a	31.00 ± 1.97 b	56.40 ± 3.63 a	48.62 ± 2.45 a	24.97 ± 1.78 c	ns	****	ns
Total biomass (g)	68.79 ± 3.02 a	65.31 ± 5.18 a	45.14 ± 1.65 b	67.20 ± 4.34 a	59.19 ± 2.68 a	33.62 ± 1.75 c	*	****	ns
Leaf area(cm^2^)	209.64 ± 2.87 c	193.54 ± 4.13 c	137.40 ± 3.48 d	418.59 ± 3.2a	421.06 ± 10.99 a	248.43 ± 4.64 b	****	****	****
Root-shoot ratio	0.27 ± 0.02 bc	0.35 ± 0.12 b	0.48 ± 0.06 a	0.19 ± 0.01 c	0.22 ± 0.03 c	0.35 ± 0.05 b	****	****	ns
Root length (cm)	94.17 ± 8.20 a	77.17 ± 4.88 ab	72.6 ± 5.43 bc	57.42 ± 3.65 cd	54.33 ± 2.73 d	49.67 ± 3.31 d	****	*	ns
	80%r	50%r	15%r	80%r	50%r	15%r			
Leaf biomass(g)	40.18 ± 2.06 ab	34.31 ± 1.53 b	21.49 ± 2.06 c	41.79 ± 2.38 a	41.96 ± 2.46 a	23.65 ± 1.04 c	*	****	ns
Stem biomass (g)	42.03 ± 1.64 a	35.05 ± 1.43 a	18.28 ± 1.91 b	36.15 ± 2.59 a	35.02 ± 3.21 a	12.22 ± 0.78 b	ns	****	ns
Wood biomass (g)	28.66 ± 1.58 a	20.44 ± 1.59 b	10.46 ± 1.24 c	22.61 ± 1.77 b	21.25 ± 2.32 b	6.12 ± 0.42 c	*	****	ns
Root biomass (g)	16.00 ± 1.29 a	15.32 ± 0.77 a	12.69 ± 1.30 a	15.96 ± 0.57 a	12.70 ± 1.00 a	8.23 ± 0.31 b	****	****	ns
Aboveground biomass (g)	81.09 ± 3.74 a	71.24 ± 3.06 a	42.19 ± 3.80 b	74.55 ± 4.20 a	72.21 ± 3.73 a	35.87 ± 1.72 b	ns	****	ns
Total biomass (g)	98.40 ± 3.26 a	84.82 ± 5.48 a	52.47 ± 3.88 b	93.91 ± 5.35 a	90.25 ± 6.25 a	44.09 ± 1.98 b	ns	****	ns
Leaf area(cm^2^)	230.85 ± 9.57 c	218.42 ± 5.22 cd	184.85 ± 16.51 d	424.54 ± 20.2 a	414.24 ± 8.13 a	336.69 ± 9.12 b	****	****	ns
Root-shoot ratio	0.19 ± 0.02 b	0.26 ± 0.03 ab	0.34 ± 0.06 a	0.21 ± 0.01 b	0.17 ± 0.01 b	0.23 ± 0.01 b	*	*	ns
Root length (cm)	90.00 ± 10.02 ab	99.67 ± 11.43 a	69.00 ± 4.64 bc	80.67 ± 6.21 ab	71.50 ± 4.59 bc	50.80 ± 3.09 c	**	*	ns

The bar indicates mean ± SE. Different letters after the mean values in the same row indicate significant difference. ANOVAS of cultivars (C), soil water content (W) and their interaction (C × W) are also indicated. *P < 0.05; **P < 0.01; ***P < 0.001; ****P < 0.0001; ns, not significant. 80%, 50%, 15% indicate control, moderate drought and severe drought treatment. 80%r, 50%r, 15%r indicate the corresponding re-watering treatment.

Except for a significant increase in O_2_^•−^ concentrations in leaves of Zhongshen1, no significant changes in free proline, MDA and H_2_O_2_ were observed under MD (**Supplementary Figure S1**). Under SD, free proline and O_2_^•−^ of both varieties significantly increased to levels as high as 8- and 2-fold respectively over those of control, whereas H_2_O_2_ level rose significantly only in Zhongshen1. Wubu accumulated higher level of free proline and lower levels of O_2_^•−^ and H_2_O_2_ than Zhongshen1. SD induced ROS accumulation of both cultivars dropped to similar levels as the control after rewatering, whereas SD plants sustained high levels of free proline after rehydration, despite that an abrupt decline was observed.

### 3.2 Variations in wood anatomy and carbohydrates

**Figure 1:**
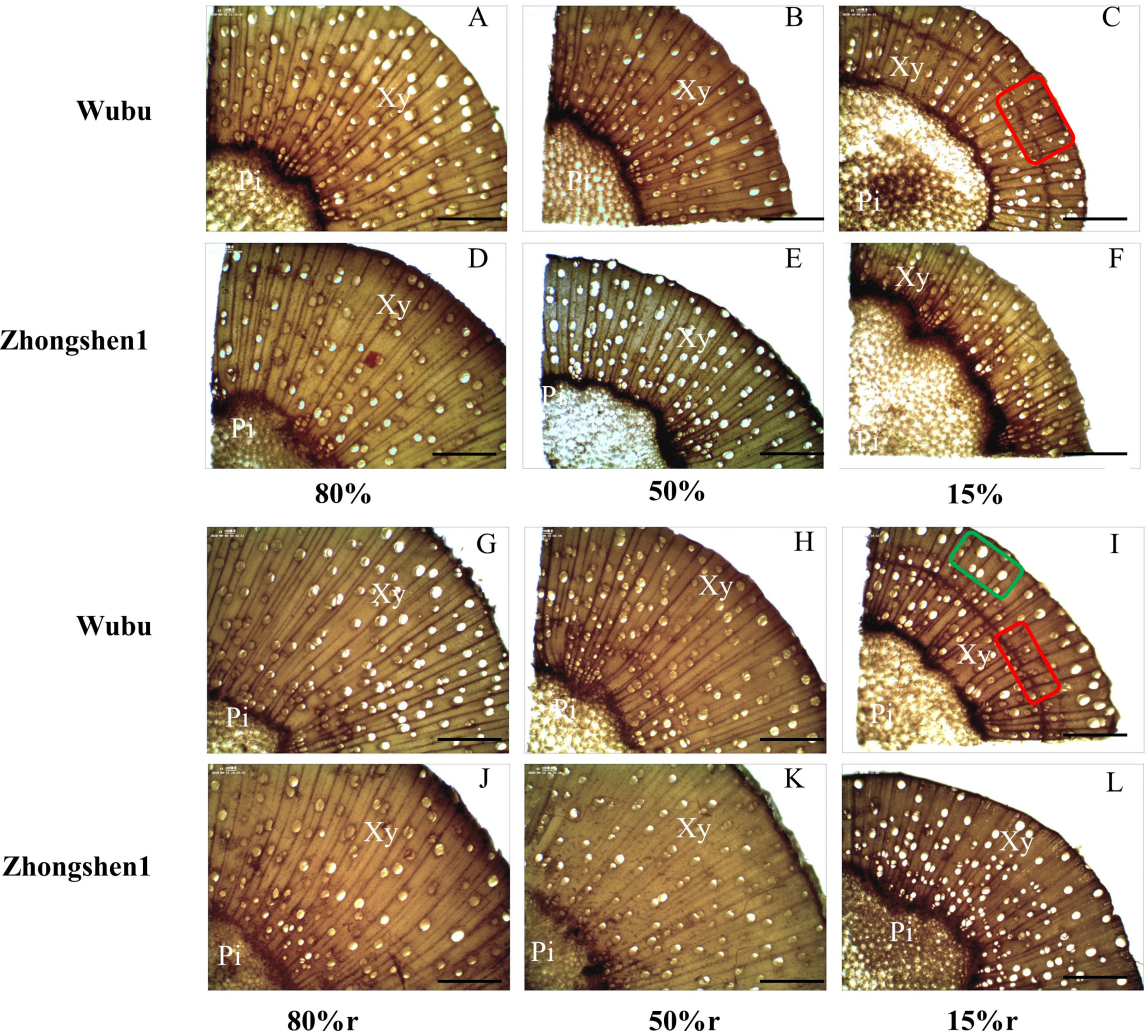
Wood cross section of 6-month-old mulberry saplings Wubu (drought-resistant) and Zhongshen1 (drought-susceptible) exposed to 80, 50 and 15% field capacity for 21 days **(A–F)** and re-watered to 80% field capacity **(G-L**, denoted as 80%r, 50%r and 15%r) for 12 days. The vessels generated during drought and re-watering are indicated in the red and green box, respectively. Images were taken at × 100 magnification. The bar at the bottom indicates 400 μm. Pi, pith; Xy, xylem.

Wood anatomy was significantly changed in response to different levels of drought stress (**Table 3**; **Figure 1**). MD and SD led to significantly decreased radius of wood and xylem in both cultivars without effecting pith width, indicating retarded radical growth (**Table 3**). MD did not cause obvious variations in vessel structures, except for decreased fiber cell length in Wubu. In contrast, SD significantly reduced the VLD, VLA, fiber cell length, and increased VF of both cultivars, with no changes in vessel element length (**Table 3**). It is noteworthy that the VLD of Wubu decreased more than that of Zhongshen1 (64.81% vs 39.33%), and the VF of Wubu was nearly 2-fold higher than that of Zhonshen1. Consistent with these, aberrant vessels were found within SD xylem produced after drought exposure (**Figure 1**). Most notably, narrow bands of highly clustered, small-diameter vessels were observed in tangential arcs of the outer layer xylem of Wubu rather than Zhongshen1. Such vessel structural modification corresponded well to the unaltered predicted hydraulic conductivity of vessels under drought stress (**Table 3**). After rehydration, the radical growth of wood was partially recovered, the size and density of the newly generated vessels returned to the condition similar to control (**Figure 1**). Although vessel frequency in SD of both cultivars after rehydration vastly dropped (especially Wubu) compared to SD, it was significantly higher than that of WWr (**Table 3**).

**Table 3:** Effects of drought and re-watering on the anatomical characteristics of wood in drought-resistant Wubu and drought-susceptible Zhongshen1 exposed to 80, 50 and 15% field capacity for 21 days and re-watered to 80% field capacity (denoted as 80%r, 50%r and 15%r) for 12 days, respectively.

Variables	Wubu			Zhongshen1			ANOVA		
	80%	50%	15%	80%	50%	15%	C	W	C × W
Vessel frequency (mm^-2^)	28.56 ± 1.16 cd	35.67 ± 2.08 c	115.28 ± 5.98 a	24.05 ± 2.62 d	27.38 ± 1.74 cd	68.28 ± 3.39 b	****	****	****
Vessel lumen length (μm)	172.02 ± 5.06 b	161.18 ± 2.76 b	157.59 ± 6.08 b	227.23 ± 4.87 a	230.60 ± 6.53 a	222.51 ± 9.85 a	****	ns	ns
Vessel lumen diameter (μm)	75.32 ± 2.37 ab	69.68 ± 1.96 b	40.45 ± 1.12 d	78.28 ± 1.79 a	73.42 ± 2.26 ab	59.87 ± 1.40 c	****	****	***
Vessel lumen area (μm^2^)	4721.48 ± 271.86 ab	4081.72 ± 245.51 b	1661.28 ± 72.07 d	5044.11 ± 229.58 a	4421.63 ± 244.70 ab	3060.31 ± 133.19 c	**	****	*
Fiber cell length (μm)	466.49 ± 7.75 a	424.32 ± 6.49 b	406.38 ± 5.90 b	423.01 ± 7.02 b	424.68 ± 6.53 b	419.29 ± 5.06 b	ns	****	****
Wood thickness (mm)	2.64 ± 0.07 b	2.43 ± 0.13 c	1.54 ± 0.02 d	3.00 ± 0.03 a	2.55 ± 0.04 bc	1.65 ± 0.06 d	**	****	ns
Radius of pith (mm)	0.71 ± 0.04 ab	0.66 ± 0.05 b	0.67 ± 0.02 b	0.82 ± 0.03 a	0.82 ± 0.02 a	0.82 ± 0.04 a	****	ns	ns
Xylem thickness (mm)	1.93 ± 0.02 b	1.76 ± 0.03 c	0.87 ± 0.01 d	2.18 ± 0.02 a	1.73 ± 0.02 c	0.83 ± 0.01 d	****	****	****
Pith area/Total area	0.07 ± 0.01 d	0.08 ± 0.01 d	0.19 ± 0.01 b	0.08 ± 0.01 d	0.10 ± 0.00 c	0.25 ± 0.01 a	****	****	*
Xylem area/Total area	0.80 ± 0.01 a	0.79 ± 0.01 a	0.65 ± 0.01 b	0.81 ± 0.01 a	0.79 ± 0.00 a	0.60 ± 0.01 c	**	****	***
Total vessel area/Total area	0.12 ± 0.01 b	0.13 ± 0.00 ab	0.16 ± 0.01 a	0.11 ± 0.01 b	0.11 ± 0.00 b	0.15 ± 0.01 a	ns	****	ns
hydraulic conductivity (×10^-6^ mm^-2^)	75.91 ± 5.89 a	65.76 ± 4.42 a	86.53 ± 22.12 a	76.74 ± 5.88 a	64.47 ± 5.38 a	76.06 ± 5.71 a	ns	ns	ns
	80%r	50%r	15%r	80%r	50%r	15%r			
Vessel frequency (mm^-2^)	29.83 ± 2.04 c	32.69 ± 2.19 c	66.08 ± 2.42 a	22.53 ± 1.74 d	23.51 ± 1.71 d	53.41 ± 2.32 b	****	****	ns
Vessel lumen length (μm)	180.80 ± 4.75 b	185.78 ± 5.54 b	182.72 ± 6.09 b	216.73 ± 7.37 a	212.56 ± 6.54 a	210.83 ± 0.29 a	****	ns	ns
Vessel lumen diameter (μm)	74.6 ± 1.99 ab	69.55 ± 2.42b	57.73 ± 0.79 c	78.85 ± 1.08 a	77.30 ± 1.55 a	59.12 ± 2.09 c	**	****	ns
Vessel lumen area (μm^2^)	4691.63 ± 220.29 ab	4108.52 ± 254.73 b	3148.86 ± 97.82 c	5181.86 ± 157.73 a	4897.29 ± 187.22 a	3062.28 ± 191.90 c	*	****	ns
Fiber cell length (μm)	480.35 ± 16.01 a	441.93 ± 12.64 b	408.47 ± 11.75 b	423.12 ± 12.31 b	423.21 ± 3.61 b	418.07 ± 3.00 b	*	*	*
Radius of the wood (mm)	3.69 ± 0.03 b	2.84 ± 0.13 d	1.84 ± 0.03 e	3.99 ± 0.10 a	3.38 ± 0.07 c	1.98 ± 0.03 e	****	****	*
Radius of pith (mm)	0.72 ± 0.04 c	0.67 ± 0.06 c	0.65 ± 0.02 c	0.94 ± 0.07 a	0.86 ± 0.01 ab	0.77 ± 0.03 bc	****	*	ns
Xylem length (mm)	2.97 ± 0.02 b	2.17 ± 0.04 d	1.2 ± 0.01 e	3.05 ± 0.03 a	2.53 ± 0.03 d	1.22 ± 0.01 e	****	****	****
Pith area/Total area	0.04 ± 0.00 d	0.06 ± 0.01 cd	0.13 ± 0.01 b	0.06 ± 0.01 cd	0.07 ± 0.01 c	0.15 ± 0.01 a	*	****	ns
Xylem area/Total area	0.82 ± 0.01 a	0.81 ± 0.01 a	0.70 ± 0.01 b	0.83 ± 0.01 a	0.83 ± 0.01 a	0.71 ± 0.02 b	ns	****	ns
Total vessel area/Total area	0.14 ± 0.01 b	0.14 ± 0.01 b	0.18 ± 0.01 a	0.11 ± 0.00 c	0.11 ± 0.01 c	0.14 ± 0.01 b	****	****	ns
hydraulic conductivity (×10^-6^ mm^-2^)	76.68 ± 4.43 a	70.96 ± 4.46 a	83.61 ± 5.84 a	78.09 ± 6.19 a	68.02 ± 5.87 a	69.21 ± 7.76 a	ns	ns	ns

The bar indicates mean ± SE. Different letters after the mean values in the same row indicate significant difference. ANOVAS of cultivars (C), soil water content (W) and their interaction (C × W) are also indicated. *P < 0.05; **P < 0.01; ***P < 0.001; ****P < 0.0001; ns, not significant. 80%, 50%, 15% indicate control, moderate drought and severe drought treatment. 80%r, 50%r, 15%r indicate the corresponding re-watering treatment.

**Figure 2:**
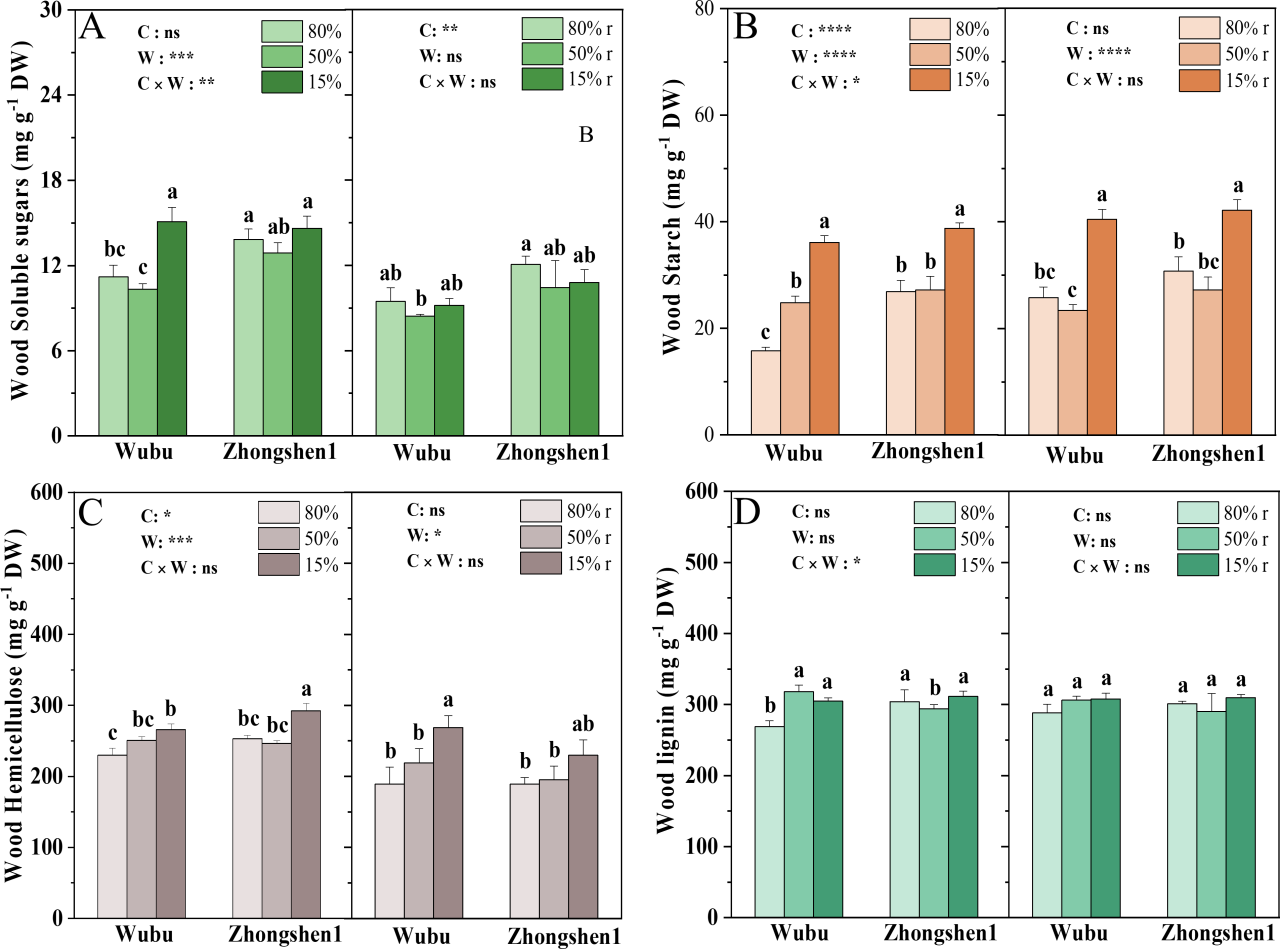
Concentrations of total soluble sugar **(A)**, starch **(B)**, hemicellulose **(C)** and lignin **(D)** in the wood in drought-resistant Wubu and drought-susceptible Zhongshen1 exposed to 80, 50 and 15% field capacity for 21 days and re-watered to 80% field capacity (denoted as 80%r, 50%r and 15%r) for 12 days, respectively. The bar indicates mean ± SE (n = 6). Different letters on the bars indicate significant difference. ANOVAs of cultivar (C), soil water content (W), and their interaction (C × W) are also indicated. *P < 0.05; **P < 0.01; ***P < 0.001; ****P < 0.0001; ns, not significant.

MD had no significant effects on the soluble sugars and starch content in the wood of both cultivars, except for a significant increase in starch content in Wubu (**Figures 2A, B**). SD led to significant increase in the starch content of both cultivars and soluble sugar content of Wubu (**Figures 2A, B**). The concentrations of soluble sugar were recovered while those of starch did not after rewatering. The concentrations of hemicellulose were unchanged under MD and rewatering in both cultivars, whereas increased significantly under SD and recovered only in Zhongshen1 after rehydration (**Figure 2C**). The concentrations of lignin in wood increased in Wubu and decreased in Zhongshen1 under MD, whereas only Wubu showed elevated lignin under SD. No differences were found after rewatering in both cultivars (**Figure 2D**).

### 3.3 Non-structural carbohydrates and phytohormones of cambial zone

**Figure 3:**
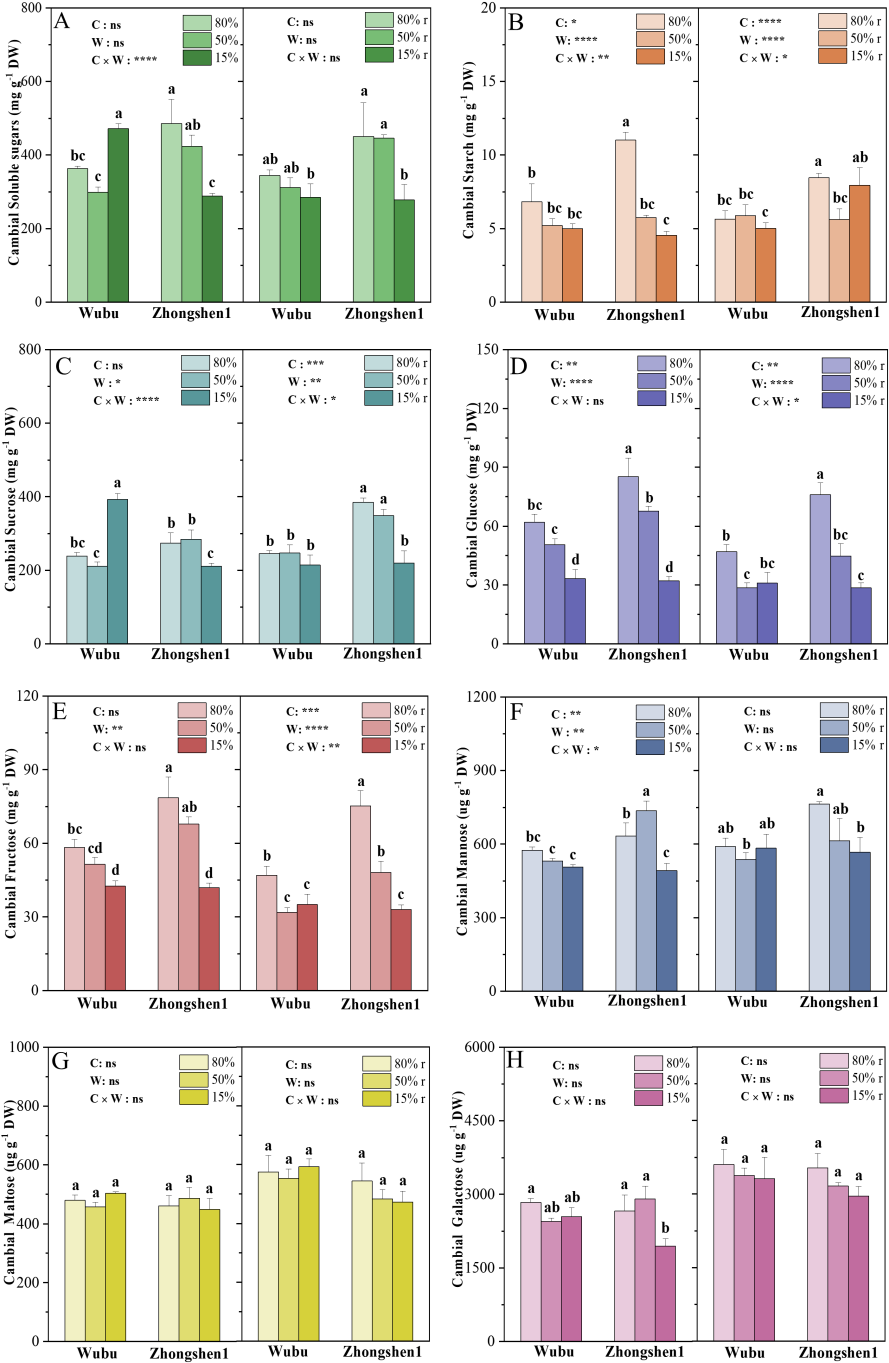
Concentrations of total soluble sugar **(A)**, starch **(B)**, sucrose **(C)**, glucose **(D)**, fructose **(E)**, mannose **(F)**, maltose **(G)** and galactose **(H)** in the cambial zone of stem in drought-resistant Wubu and drought-susceptible Zhongshen1 exposed to 80, 50 and 15% field capacity for 21 days and re-watered to 80% field capacity (denoted as 80%r, 50%r and 15%r) for 12 days. The bar indicates mean ± SE (n = 6). Different letters on the bars indicate significant difference. ANOVAs of cultivar (C), soil water content (W), and their interaction (C × W) are indicated. *P < 0.05; **P < 0.01; ***P < 0.001; ****P < 0.0001; ns, not significant.

MD significantly reduced the starch content in the cambial zone of Zhongshen1, while SD led to increased concentrations of soluble sugars in Wubu and decreased levels of soluble sugars and starch in Zhongshen1 (**Figures 3A, B**). After rehydration, the soluble sugars of Wubu fully recovered. The starch content of both varieties previously exposed to SD recovered to the WWr level. Considering the opposite changes in soluble sugars between Zhongshen1 and Wubu under SD, concentrations of several simple soluble sugars were determined (**Figures 3C–H**). The variations of sucrose were highly consistent with the total soluble sugars (**Figure 3C**). After rehydration, the sucrose content of Wubu decreased to the level of control, but that of Zhongshen1 did not. The concentrations of glucose, fructose and mannose decreased more in Zhongshen1 than Wubu under SD, which were not recovered after rehydration (**Figures 3D–F**). The concentrations of maltose and galactose did not respond to water shortage in both varieties (**Figures 3G, H**).

**Figure 4:**
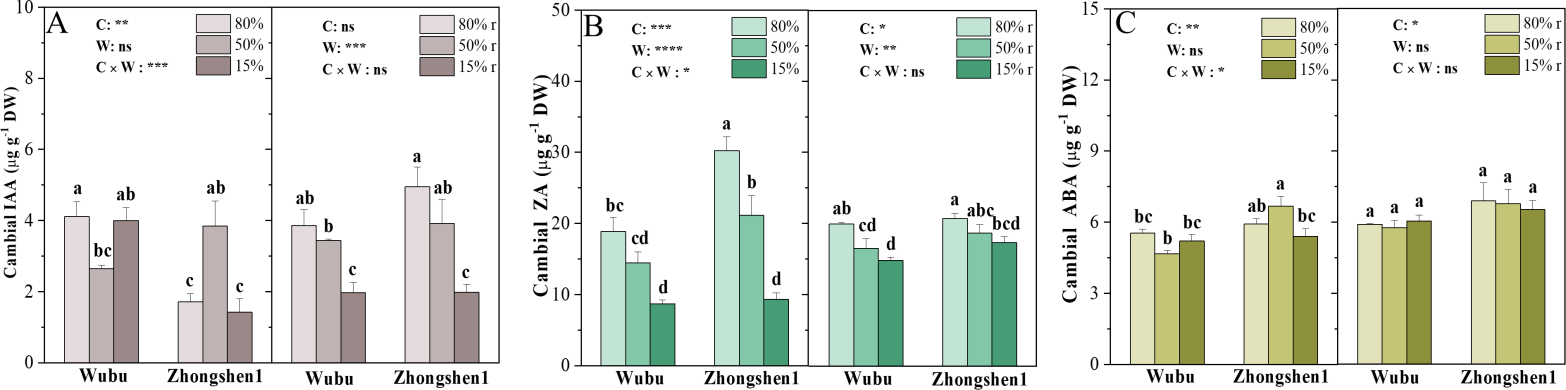
Concentrations of IAA **(A)**, ZA **(B)** and ABA **(C)** in the cambial zone of stem in drought-resistant Wubu and drought-susceptible Zhongshen1 exposed to 80, 50 and 15% field capacity for 21 days and re-watered to 80% field capacity (denoted as 80%r, 50%r and 15%r) for 12 days. The bar indicates mean ± SE (n = 6). Different letters on the bars indicate significant difference. ANOVAs of cultivar (C), soil water content (W), and their interaction (C × W) are also indicated. *P < 0.05; **P < 0.01; ***P < 0.001; ****P < 0.0001; ns, not significant.

The concentrations of IAA increased in Wubu and decreased in Zhongshen1 under MD, while remained comparable to the control under SD (**Figure 4A**). Noteworthily, Wubu had higher levels of IAA under WW and SD. Both varieties exhibited continuously decreased ZA as water supply reduced (**Figure 4B**). IAA and ZA were partially or fully recovered after rehydration. No changes were found for ABA levels in response to drought and rewatering (**Figure 4C**).

### 3.4 Transcriptome sequencing analysis of cambial zone

**Figure 5:**
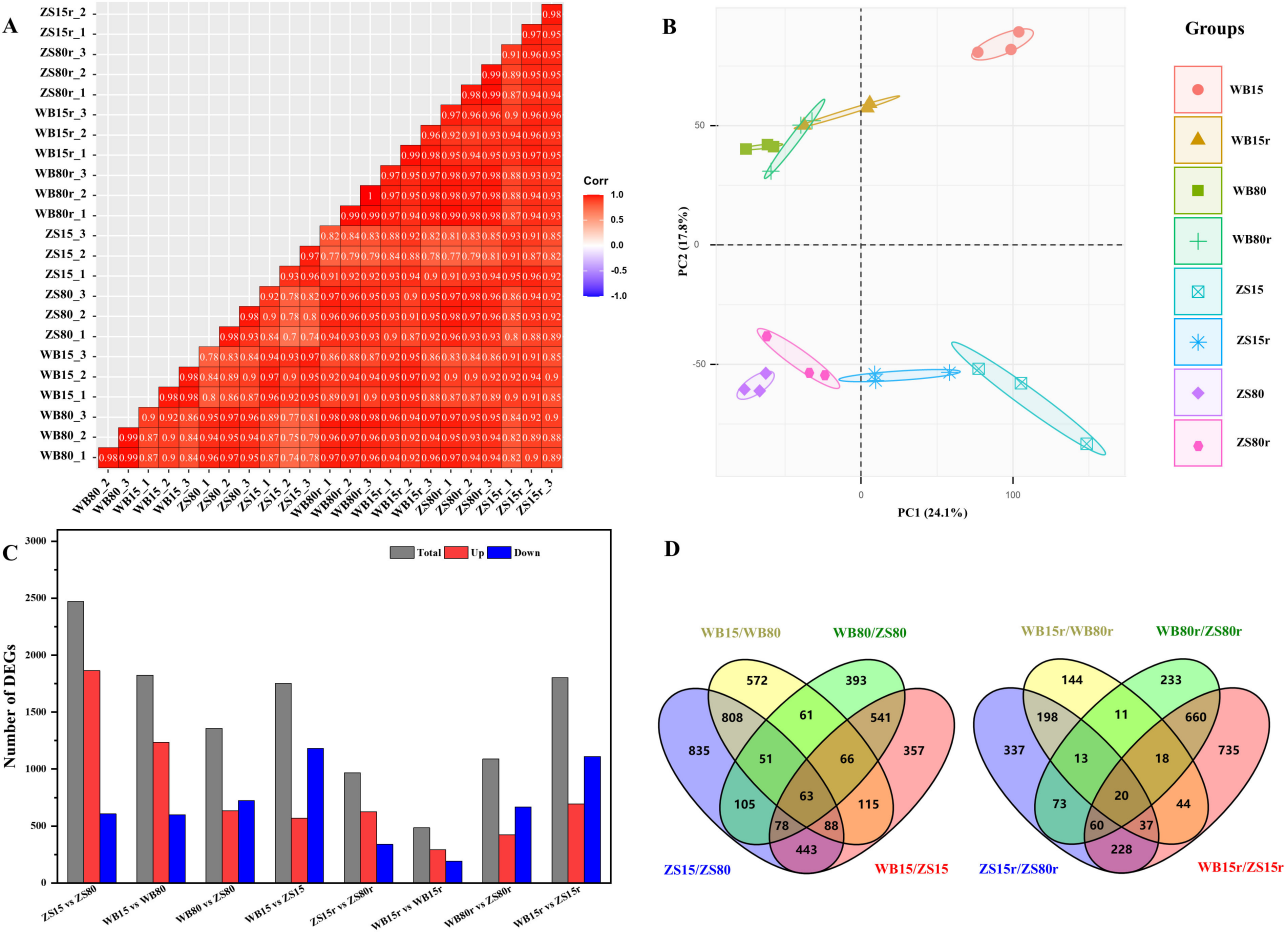
Overview of the RNA-seq results in the cambial zone of drought-resistant Wubu and drought-susceptible Zhongshen1 exposed to 80 and 15% field capacity for 21 days and re-watered to 80% field capacity (denoted as 80%r and 15%r) for 12 days. Pearson correlation **(A)**, PCA plot **(B)**, number of DEGs in eight comparison groups **(C)** and Venn diagram of the overlapped DEGs **(D)** are presented. The color code from bule to red represents the Pearson value from -1 to 1.

Given the distinct wood anatomy of the examined varieties under SD, comparative transcriptome was used to analyze the underlying transcriptional regulations of developing cambium in response to drought (**Figure 5**). 24 samples were sequenced and totally obtained *ca*. 25.92 Gb of raw data. Q20 ≥ 97.21 and Q30 ≥ 92.22 were identified in all samples. An average of 94.4% were uniquely matched to the reference genomes (**Supplementary Table S2**). Pearson correlation analysis showed that the three biological replicates had good consistency (R > 0.93, **Figure 5A**), as evidenced by the clustered samples within groups in PCA based on transcriptomic profiles (**Figure 5B**). Variability observed between water regimes were mostly represented along PC1, whereas variability between cultivars was clearly separated by PC2. SD induced 2471 DEGs in Zhongshen1, of which 1864 were upregulated and 607 were downregulated. Wubu had 1235 upregulated and 589 downregulated DEGs under SD. After rehydration, the number of up- and down-regulated genes in Wubu significantly decreased, with only 293 and 192 genes, respectively. The DEGs of Zhongshen1 reduced to 966 after rewatering (**Figure 5C**; **Supplementary Table S3**). Furthermore, 1010 and 268 DEGs were common DEGs in response to drought and rewatering in both cultivars, respectively. Among these common DEGs, 63 and 20 DEGs were cultivar-specific (**Figure 5D**; **Supplementary Table S4**). KEGG analysis found that most drought-induced DEGs in Wubu were enriched in protein processing, starch and sucrose metabolism pathways, while motor proteins and diterpenoid biosynthesis pathways were enriched in Zhongshen1. The drought-responsive common DEGs were significantly enriched in folding, sorting and degradation pathway, whereas environmental adaptation, membrane transport and lipid metabolism were enriched under rewatering (**Supplementary Table S5**). The GO enrichment analysis showed that drought responsive common DEGs were significantly involved in terms such as response to salt stress, oxidative stress, hydrogen peroxide, protein maturation, DNA binding, and hydrolase activity (**Supplementary Figure S2A**). After rehydration, similar terms were still significantly enriched (**Supplementary Figure S2B**).

### 3.5 Key gene modules for drought resistance and recovery

**Figure 6:**
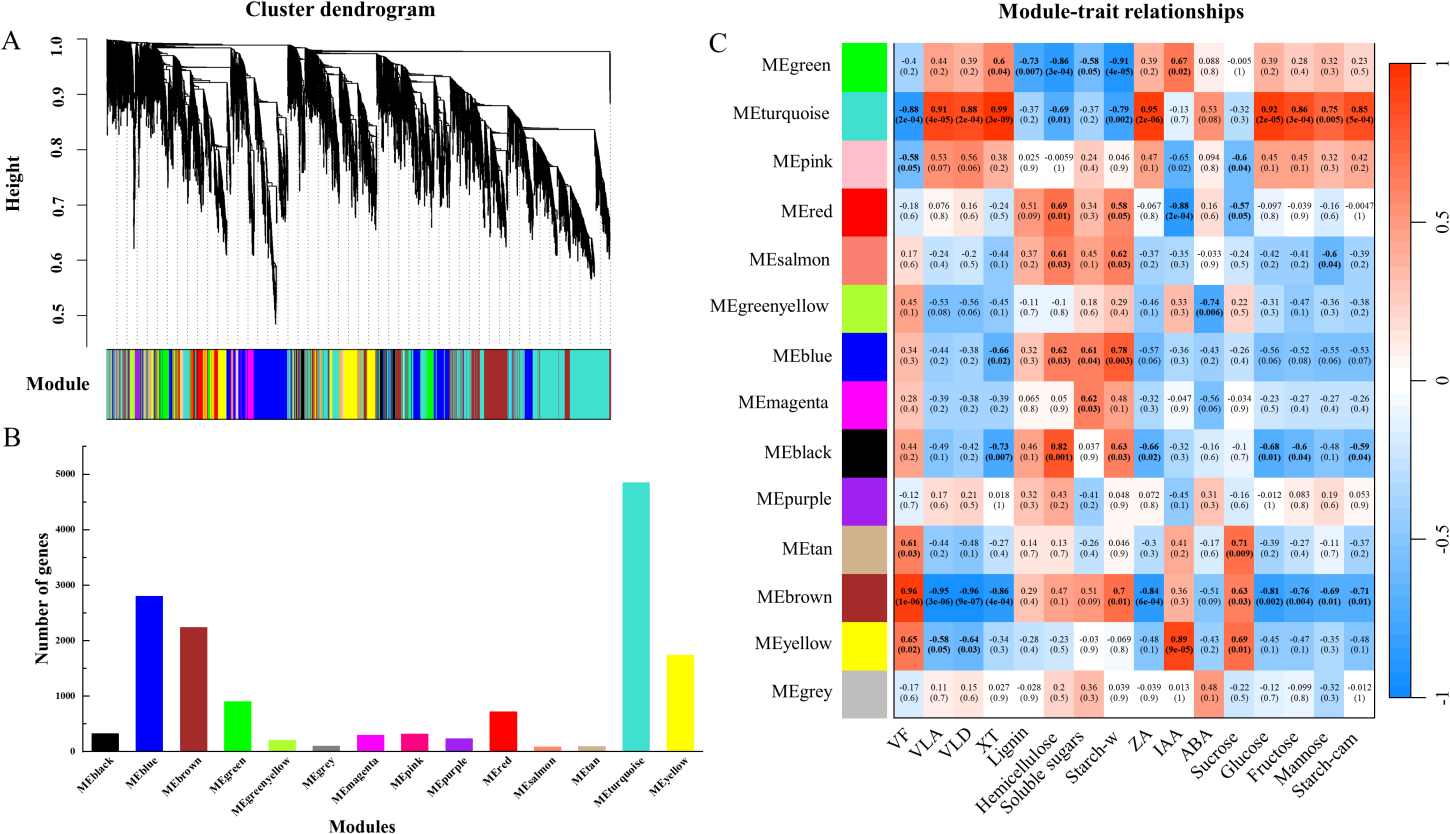
Key gene modules for drought resistance traits (xylem anatomy and the physiological responses of cambium and xylem) screened by WGCNA. Cluster dendrogram of expressed genes based on WGCNA analysis **(A)**, the number of genes in each module **(B)** and the correlation analysis between gene modules and traits **(C)** are shown. Pearson correlation coefficient (r) and *p* values (in brackets) are presented for each module. VF, vessel frequency; VLA, vessel lumen area; VLD, vessel lumen diameter; XT, xylem thickness; Starch-w, starch in wood; Starch-cam, starch in cambium.

In order to further explore the relationship between traits and DEGs, WGCNA analysis was performed with genes (FPKM value > 5) expressed under drought and rewatering. The tree of gene clusters was successfully constructed, and 14 stable expression modules were obtained (**Figure 6A**). The number of genes in each module varied greatly, ranging from 84 to 4846 (**Figure 6B**). Module-trait relationships were analyzed to find the key modules related to the vessel structural and physiological parameters of wood and cambial zone (**Figure 6C**). MEturquoise was negatively correlated with VD, starch and hemicellulose in wood, the correlation coefficients were 0.88, 0.69, 0.79. Conversely, MEturquoise were positively correlated with VLD, VLA, xylem radius, ZA, glucose, fructose, mannose and starch in the cambial zone, the correlation coefficients were 0.91, 0.88, 0.99, 0.95, 0.92, 0.86, 0.75, 0.85. Additionally, 18 stable modules were obtained for rewatering, among which MEgreen was also negatively correlated with VD, hemicellulose and starch in wood, and positively correlated with VLA, VLD, XW, ZA, IAA, sucrose, fructose and glucose (**Supplementary Figure S3**).

**Figure 7:**
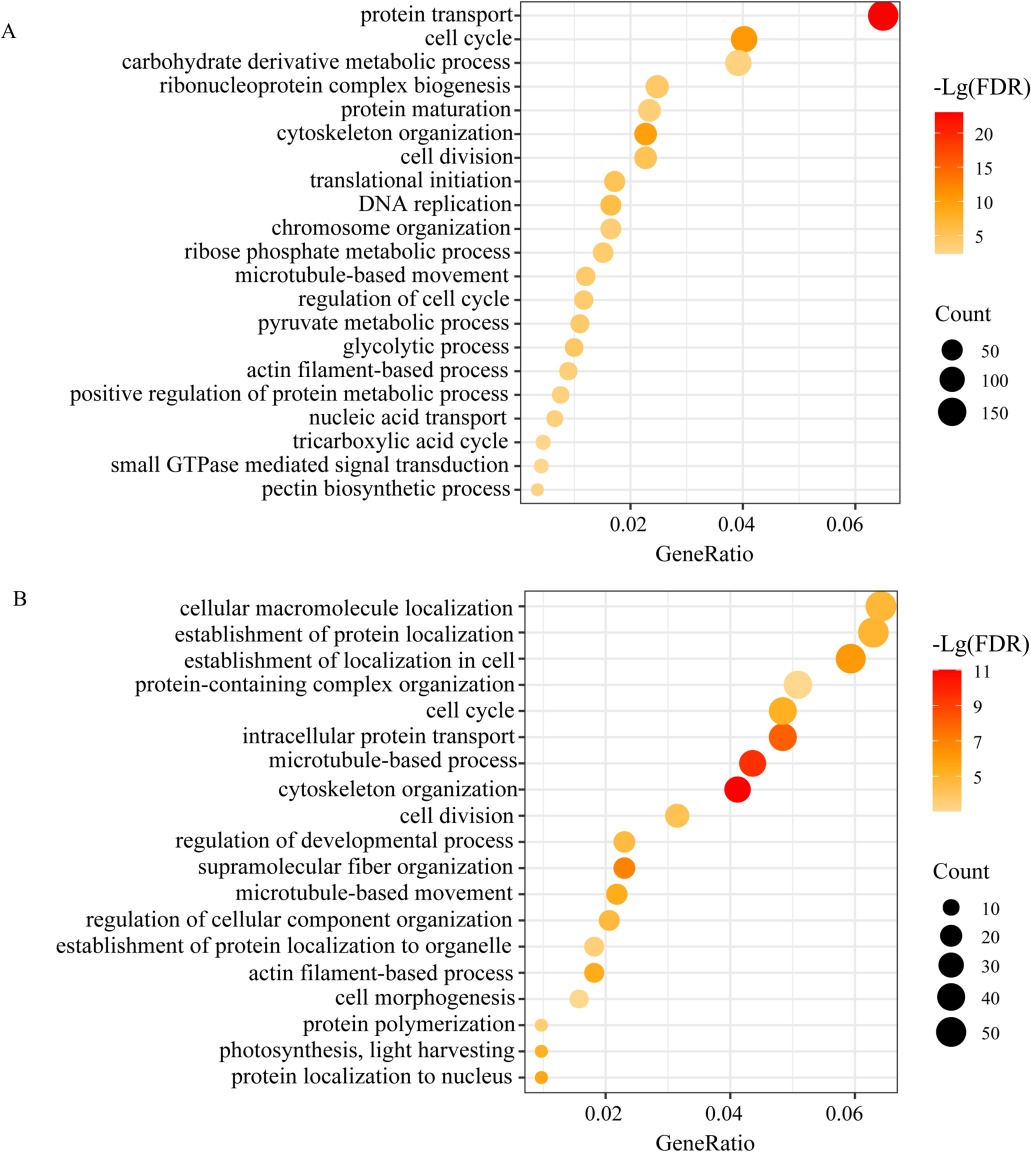
Biological processes in GO enrichment analysis of the turquoise module under drought **(A)** and the green module after rewatering **(B)**.

To obtain information on the functions that were correlated with the physiological and or anatomical traits, drought-responsive genes of MEturpuoise (noted as MEturpuoise-D) and rewatering responsive genes of MEgreen (noted as MEgreen-R) were further categorized by GO and KEGG enrichment analysis (**Figure 7**, **Supplementary Table S7**). MEturpuoise-D was enriched and annotated into “biological processes”, “cell component”, and “molecular functions”. The predominant terms in the biological process group were protein localization (GO: 0008104), intracellular transport (GO: 0046907), cell cycle (GO: 0007049), cytoskeleton organization (GO: 0007010), microtubule-based process (GO: 0007017) and proteolysis involved in protein catabolic process (GO: 0051603); membrane coat (GO: 0030117) and cytoskeleton (GO: 0005856) were the most enriched terms in the cellular component group. In the molecular function ontology, the highest percentages of DEGs belonged to the cytoskeletal protein binding (GO: 0005196), structural molecule activity and translation regulator activity (GO: 0045182) groups. KEGG analysis showed that translation, cell motility, carbohydrate metabolism, folding, sorting and degradation were significantly enriched pathways (**Supplementary Table S7**). MEbrown-D was enriched in transcription. As expected, MEgreen-R and MEgreenyellow-R corresponded well to MEturquoise-D and MEbrown-D as they showed similar GO and KEGG enrichment results (**Supplementary Table S7**), indicating partially recovery of transcription.

### 3.6 DEGs associated with xylem plasticity

**Figure 8:**
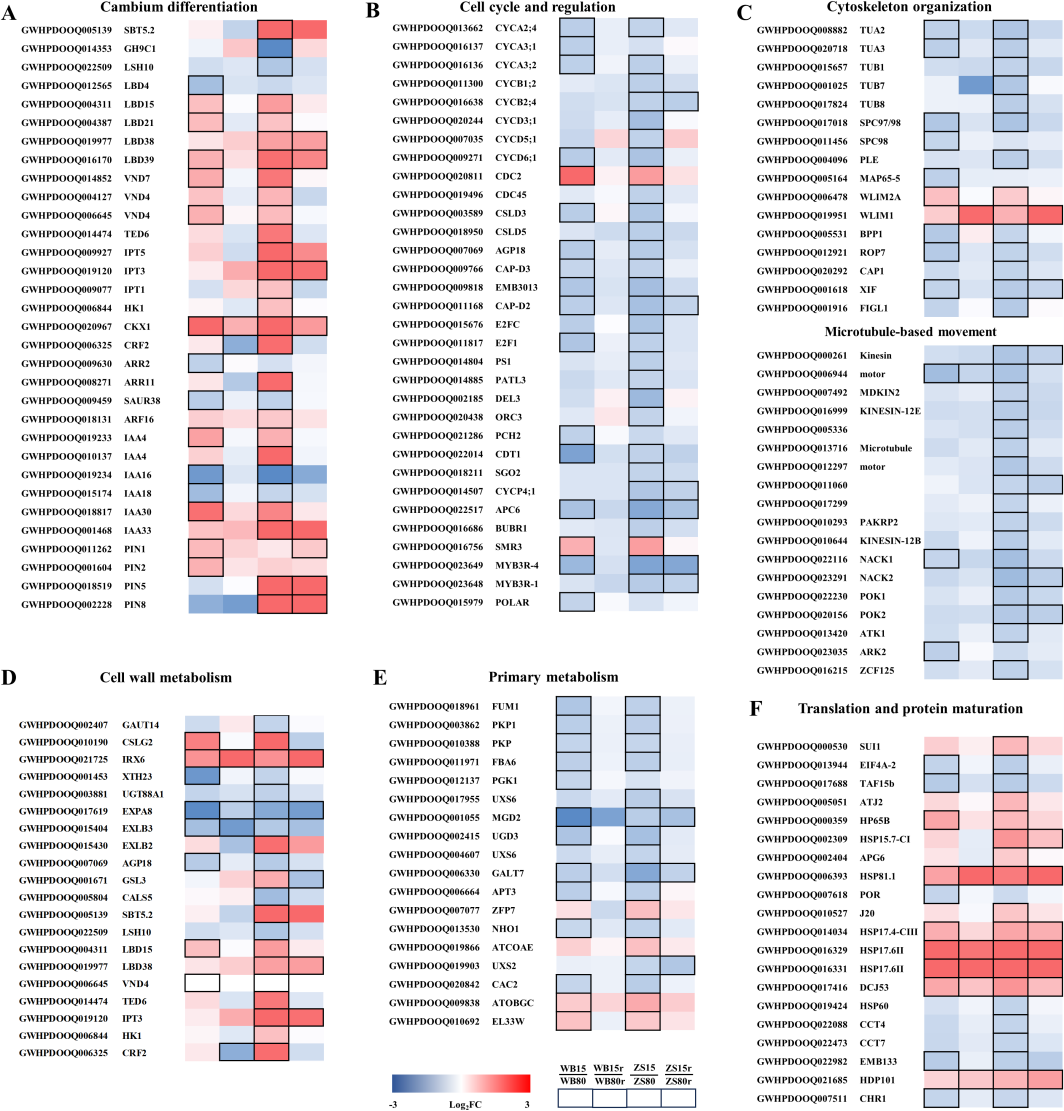
Selected candidate DEGs from WGCNA analysis involved in cambium differentiation **(A)**, cell cycle and regulation **(B)**, cytoskeleton organization **(C)**, cell wall metabolism **(D)**, translation and protein maturation **(E)** and primary metabolism **(F)** in response to severe drought stress in mulberry. The changes of transcriptional expression levels in the four comparison groups are illustrated with colored boxes. The boxes from the leaf to right represent WB15 vs. WB80, WB15r vs. WB80r, ZS15 vs. ZS80, and ZS15r vs. ZS80r. The color code from blue to red indicates the fold changes between -3 and 3. Bordered boxes indicate significantly expressed genes in either of the four comparison groups.

The xylem anatomical analysis suggested a reprogramming of cambium differentiation responding to water supply. Therefore, we searched for candidate regulators of cambium development based on functional annotations. Among the drought-responsive DEGs determined in the cambial zone, 32 genes with predicted roles in the regulation of cambium differentiation were screened, which were mostly up-regulated under SD (**Figure 8A**). Specifically, *LOB DOMAIN-CONTAINING PROTEIN 15* (*LBD15*), *LBD21*, *LBD39*, *VASCULAR-RELATED NAC DOMAIN 7* (*VND7*) and *VND4* were significantly up-regulated in both cultivars, with higher fold changes in Zhongshen1 than Wubu. Otherwise, several genes including *SUBTILISIN-LIKE PROTEASE 5.2* (*SBT5.2*), *LBD38*, one of the two *VND4* genes and *TRACHEARY ELEMENT DIFFERENTIATION-RELATED 6* (*TED6*) were only upregulated in Zhongshen1. Conversely, *GLYCOSYL HYDROLASE 9C1* (*GH9C1*) and *LIGHT-DEPENDENT SHORT HYPOCOTYLS 10* (*LSH10*) were downregulated only in Zhongshen1. *LBD4* showed downregulation in Wubu only. Additionally, genes including *ISOPENTENYLTRANSFERASE 1* (*IPT1*), *IPT3*, *IPT5*, *HISTIDINEKINASE 1* (*HK1*), *CYTOKININ RESPONSE FACTOR 2* (*CRF2*), *ARABIDOPSIS RESPONSE REGULATOR 11* (*ARR11*) involved in cytokinin biosynthesis and signaling were only upregulated in Zhongshen1, except *CYTOKININ OXIDASE/DEHYDROGENASE 1* (*CKX1*) significantly upregulated in both cultivars. Genes involved in auxin biosynthesis and signaling were consistently up-regulated in both cultivars. Two of the four *PIN* showed down-regulation in Wubu but upregulation in Zhongshen1.

Since vessel density increased dramatically in response to severe drought, genes directly participating cell cycle and regulation were identified. Interestingly, except *CELL DIVISION CONTROLLER 2* (*CDC2*) and cyclin-dependent kinase inhibitor *SMR3*, all the genes were significantly downregulated in both cultivars, while the repression was stronger in Zhongshen1 than in Wubu (**Figure 8B**). This was also the case for the expression pattern of genes involved in cytoskeleton organization and microtubule-based movement (**Figure 8C**). Kinesin motor related genes almost did not respond to severe drought in Wubu, but were significantly downregulated in Zhongshen1. Moreover, the expression levels of genes related to cell wall establishment were examined (**Figure 8D**). We have identified the downregulation of two *GALACTURONOSYLTRANSFERASE* genes *GAUT14* and *15* related to pectin biosynthesis and one *UDP-GLUCOSYL TRANSFERASE 88A1* (*UGT88A1*) gene involved in lignin biosynthesis. *GLUCAN SYNTHASE-LIKE 3* (*GSL3*), *GSL*4, *GSL*12 were upregulated and one *CALLOSE SYNTHASE* gene *CALS5* was downregulated. In contrast, *CELLULOSE SYNTHASE-LIKE G2* (*CSLG2*) and *CSLC6* participating cellulose biosynthesis were down- and upregulated, respectively. *IREGULAR XLYLEM 6* (*IRX6*) modulating cellulose synthase velocity was upregulated. Most of the genes associated with cell wall loosening or reorientation such as *XYLOGLUCAN ENDOTRANSGLUCOSYLASE/HYDROLASE* (*XTH23*), *EXPANSIN A* and *EXPANSIN LIKE B* (*EXLB*) were downregulated.

Several genes including two pyruvate kinase genes, *FRUCTOSE-BISPHOSPHATE ALDOLASE* (*FBA6*), *PHOSPHOGLYCERATE KINASE 1* (*PGK1*) and *FUMARASE 1* (*FUM1*) involved in tricarboxylic acid cycle and glycolytic process were downregulated without obvious cultivar differences. Conversely, *ARABIDOSIS OBG GTPASE* (*ATOBGC*) and *RIBOSOMAL PROREIN EL33W* were upregulated in both cultivars. Except for *ZINC FINGER PROTEIN 7* (*ZFP7*) and a dephospho-CoA kinase gene *ATCOAE*, all the detected DEGs related to carbohydrate derivative metabolic process were downregulated, whereas more genes with higher transcriptional fold changes were found in Zhongshen1. Additionally, most genes involved in protein maturation, particularly *HEAT SHOCK PROTEIN* (*HSP*), were significantly upregulated (**Figure 8F**).

### 3.7 qRT-PCR validation of RNA-seq data

To clarify the accuracy of the RNA-seq data, 15 representative genes were selected for qRT-PCR. A high correlation between the results obtained from RNA-seq and RT-qPCR was observed, supporting the RNA-seq results (**Supplementary Figure S4**).

## 4 Discussion

In this study, moderate drought did not cause growth inhibition or evoke physiological responses in leaves. However, drought-resistant Wubu accumulated higher level of free proline and produced less ROS in leaves under severe drought stress. These results are similar to our previous observations at the late stage of rapid soil dry-down (Cao et al., 2020a; Zhai et al., 2023), indicating that osmoregulation and antioxidative protection should play important roles in adapting to severe water shortage in mulberry (Carraro and Di Iorio, 2022). After rewatering, photosynthetic gas exchange and growth parameters were partially recovered while ROS generation was completely recovered in both cultivars. The biomass production of non-leafy organs of Wubu were higher than that of Zhongshen1. Therefore, Wubu is also superior in terms of rapid recovery after re-watering, which is important for reducing post-drought yield loss in economic trees (Abid et al., 2018).

### 4.1 How do xylem anatomy and wood carbohydrates respond to soil water availability in mulberry?

Xylem anatomy, a major determinant of stem hydraulic properties, can be greatly influenced by climate conditions (Rodríguez-Ramírez et al., 2022). Narrow conduits are assumed to be more resistant than wide ones to embolism resulting from significantly lower water potential in the xylem under drought, although some arguments still exist (Lens et al., 2022). In this work, we found significantly increased number of narrower vessels in both cultivars under severe drought stress, while moderate drought only led to similar but slight changes in vessel size and density. Similar structural adjustments under strong drought were also observed in poplar (Yu et al., 2021), suggesting that the magnitude of the changes depend on the severity of drought stress (Hacke et al., 2017). As a consequence, such plastic adjustment enabled comparable predicted stem hydraulic conductivity of both cultivars under different drought intensity. Unsurprisingly, the two vessel properties recovered back quickly after rewatering for MD plants, but did not completely recover for SD plants in both cultivars. These results suggest that stem xylem plasticity is reversible and strongly shaped by soil water status, thereby optimally coordinating the trade-offs between hydraulic efficiency and safety under changing environment (Bauerle et al., 2011; Li et al., 2024a). Indeed, drought-induced xylem anatomical plasticity has been documented among different organs or different drought intensities in various tree species from angiosperm to gymnosperm, but the variations in tracheid density often present opposite patterns, being either increased or decreased (Li et al., 2021; Lens et al., 2022; Franklin et al., 2023). This might highlight the importance roles of vessel lumen diameter rather than vessel density in endowing xylem embolism resistance under drought stress (Levionnois et al., 2021). Noteworthily, the xylem structural adjustments under drought or rewatering in Wubu were more pronounced than in Zhongshen1, as illustrated by the compactly distributed smaller vessels produced during drought and their disappearance after rewatering in wood cross section. The stronger xylem plasticity of Wubu was in accordance with the relatively higher values of predicted hydraulic conductivity, confirming the correlation between anatomical traits and hydraulic properties (Hajek et al., 2014).

Moreover, it has been hypothesized that refilling of embolized conduits after rewatering involves soluble NSCs, sucrose in particular, to decrease osmotic potential in the embolized xylem and promote water movement back into the embolized conduits from adjacent parenchyma cells (Bucci et al., 2003; Secchi and Zwieniecki, 2011). We also observed a significant increase of soluble sugars in wood of Wubu rather than Zhongshen1, which dropped back to the level as control after rewatering, similar to previous observations in grapevine petiole (Falchi et al., 2020) and poplar xylem sap (Pagliarani et al., 2019). This might indicate a stronger osmoregulation capacity of Wubu which endowed its higher drought resistance and recovery ability. However, the starch reserves in both cultivars also increased significantly under drought, but did not recover after rewatering. This contradicts with our findings in mulberry (Cao et al., 2020a) or other tree species (Tsamir-Rimon et al., 2021). Since drought responses are closely related to drought intensity, we could argue that the concentrations of starch in wood might mirror the repressed metabolic. In addition to NSCs, we also found increased lignin accumulation only in Wubu under SD. Lignin deposition requires a high investment of carbon resources. It can enhance cell wall stiffness, protect membrane integrity and prevent cell collapse due to dehydration (Le Gall et al., 2015). The increase of lignin has been reported in other tree species under drought stress (Barker-Rothschild et al., 2023; Dai et al., 2024). The higher lignin deposition by activating the expression of lignin biosynthetic genes improved drought tolerance of grapevine (Tu et al., 2020). This could partially explain the higher drought resistance of Wubu.

Variations in xylem anatomy and wood carbohydrates across the two cultivars showed similar patterns at different scales, demonstrating that xylem plasticity is an important strategy for mulberry trees to confront severe drought. The stronger drought resistance of Wubu should be closely associated with the higher soluble sugars and lignin deposition under drought stress.

### 4.2 Which genes or gene clusters are involved in regulating cambium activity to modulate xylem plasticity under drought and rewatering?

In this work, severe drought significantly reduced stem diameter increments in both cultivars. This coherently matched the decline of xylem thickness, indicating decreased cambium activity and secondary growth frequently detected in trees under drought (Gryc et al., 2012; Dai et al., 2024). Specifically, the emergence of increased number of newly produced narrower vessels also dictates changes in cambium activity and reprogramming of cell division and differentiation during vessel development (Fischer et al., 2019).

From the perspective of cambium activity, we expect that phytohormones auxin, cytokinin and their crosstalk might play essential roles. Auxin is a well-established positive regulator of cambial cell proliferation and elongation through complicated signaling networks (Immanen et al., 2016; Luo et al., 2018). The maintenance of cambium activity relies on the auxin maxima at the xylem side of the vascular cambium (Smetana et al., 2019). Although the auxin levels across different cell types were not assayed, the concentrations of IAA in the cambial zone seemingly did not respond to severe drought in both cultivars, coinciding with the absence of any genes involved in auxin biosynthesis or degradation. Interestingly, the transcriptional expressions of endoplasmic reticulum localized *PIN5* and *PIN8* that belong to “short” PIN (Zhou and Luo, 2018) in Zhongshen1 were significantly upregulated. Otherwise, long PINs residing on the plasma membrane including *PIN1* and *PIN2* were only upregulated in Wubu, indicating active auxin efflux. Short PINs mainly facilitate intracellular auxin compartmentalization and homeostasis while long PINs directly facilitate intercellular transport (Mravec et al., 2009). Anyway, such auxin redistribution within or between cells activated the downstream signaling pathway. The differential expressions of *PIN* members imply that vessel development under drought stress is more associated with auxin transport (Junghans et al., 2004; Johnson et al., 2018). One *SAUR* gene was downregulated in both cultivar, this could partially explain the smaller vessel diameter because SAURs play a central role in auxin-induced acid growth in the process of cell division (Stortenbeker and Bemer, 2018). It is noteworthy that the auxin levels in Wubu were always higher than those in Zhonghsen1 regardless of water conditions, indicating inherently higher cambium activity of Wubu than Zhongshen1.

Cytokinin is another key player in influencing cambium activity and positively regulating mitotic cell division (Schaller et al., 2014). Cell division is tightly regulated by cell cycle, which can be divided into four distinct phases: Gap 1 (G1), Synthesis (S), Gap2 (G2) and M (Mitosis). We found decreased cytokinin concentrations indiscriminately in both cultivars as the stress level intensified, implying reduced cambium activity (Immanen et al., 2016). The enhanced cytokinin biosynthesis and degradation and activated signaling in Zhongshen1 indicate more intensive transcriptional responses in cytokinin metabolism and signaling. These results are partially contradictory with previous studies which observed suppressed cytokinin activities under drought stress (Paul et al., 2018), which might be ascribed to the crosstalk with enhanced auxin homeostasis and signaling or other regulators such as jasmonate (Hu et al., 2024). In *Arabidopsis*, it has been well recognized that cytokinin activates cell division through induction of CYCD3 at the G1-to-S transition (Riou-Khamlichi et al., 1999). Correspondingly, three *CYCD* genes were actively repressed only in Zhongshen1 under drought and recovered after rewatering, including the rate-limiting *CYCD3;1*. However, *CYCD3;1* of Wubu did not respond to water availability. Previous studies demonstrate that *CYCD3;1* can be transcriptionally upregulated in response to sucrose, the level of *CYCD3;1* falls rapidly on sucrose depletion (Planchais et al., 2004). This nicely explained the contrasting responses because Wubu sustained higher level of sucrose than Zhongshen1, reinforcing the importance of sugar sensing and signaling in facilitating cell cycle (Rawat and Laxmi, 2024).

*MYB3R1* and *MYB3R4* enriched in GO term “cell cycle regulation” were downregulated in Zhongshen1, while only *MYB3R4* was repressed in Wubu with a lower fold change. MYB3R proteins are important transcriptional regulators at G2-to-M transition (Berckmans and De Veylder, 2009). *MYB3R1* and *MYB3R4* are the only two out of the five MYB3R genes highly expressed in shoot apical meristem of *Arabidopsis*, playing essential roles in activating the transcriptional cascade of late cell cycle genes to execute cell mitosis and cytokinesis under the regulation of cytokinin (Yang et al., 2021). MYB3R4 can bind to the mitosis-specific activator (MSA) elements in the promoters of A- and B-type cyclin which are previously reported as important regulators of the G2-M cell cycle checkpoint in dividing cells of *Arabidopsis* (Olszak et al., 2019), consistent with the observed downregulation of several representative cyclins such as *CYCB1;2* and *CYCB2;4* in this work. Loss of *MYB3R* results in downregulation of a subset of late cell cycle genes (Kobayashi et al., 2015). On the other hand, SIAMESE-RELATED 3 (SMR3), a cyclin-dependent kinase inhibitor was upregulated in both cultivars, supporting the assumption that mitosis was actively repressed. Members of SIAMESE-RELATED class inhibitor proteins can retard G2 progression and increase cell size in *Arabidopsis thaliana* (Yamada et al., 2022), while their specific role in vessel cell size need further investigations in mulberry and other woody trees. Additionally, we found that some other transcriptional factors appear to play roles in regulating xylem development, such as *VND7* responsible for xylem differentiation (Ramachandran et al., 2021) and *LBD4* required to keep cambial stem cells undifferentiated (Ye et al., 2021).

The ultimate size of wood cells is also associated with cell wall establishment, which balances extensibility and rigidity of cell walls (Zhu and Li, 2024). Cell wall formation relies on vesicles transport along cytoskeletal tracks of microtubule and actin filaments (Gu and Rasmussen, 2021). We observed that genes involved in cytoskeleton organization and microtubule-based movement were mostly downregulated in both cultivars, although at different scales. These two processes are closely related to cell cycle, vesicle transport, organelle mobility and cell wall formation (Zhang et al., 2023). Notably, two *WLIM* genes *WLIM2A* and *WLIM1* that regulate actin filament organization were upregulated, suggesting that microtubule and actin filaments could play different roles in cytoskeleton organization in cambial zone of mulberry under severe drought stress (Wang and Mao, 2019). Corresponding to the global repression of genes related to cytoskeleton organization, we found remodeling of cambial cell wall composition under drought stress. Elevated expressions of *IRX6* could accelerate the activity of *CES7* cellulose synthase complexes to increased cellulose level (Xue et al., 2024). Decreased pectin and expansin, increased cellulose and callose deposition, together contributing to attenuated cell wall extensibility. These results highlight the involvement of cambial cell wall flexibility in cambial cell proliferation in response to drought (Calderone et al., 2024).

Additionally, we also identified sets of genes involved in primary metabolism and protein translation and maturation which were highly correlated with METurquoise-D. The overall downregulation of the biological processes such as tricarboxylic acid cycle and glycolytic process was generally in line with the decreased starch, glucose, fructose and mannose and the increased transcriptional expressions of starch-degrading enzymes in the cambium zone of both cultivars. However, their relevance to xylem plasticity remains to be explored.

## 5 Conclusions

In this study, we investigated the relationships between stem xylem plasticity and cambium activity in response to soil water deficit and rewatering in mulberry saplings by combining anatomical, physiological and transcriptional methodologies. It is interesting that the two examined cultivars contrasting in drought resistance responded similarly by producing increased number of narrower vessels under drought. Fewer wider vessels were generated once the water supply was sufficient. However, such xylem plasticity acted at different scales in Wubu and Zhongshen1. By applying WGCNA, we found genes involved in cambium proliferation and differentiation, cell cycle, cell wall composition, cytoskeleton organization, primary metabolism and protein maturation were highly associated with vessel diameter and the sugar status of cambium. We assume that mulberry xylem plasticity in response to water availability is under the regulation of cambium activity in a complicated manner. Additionally, it is important to note that the responses of xylem plasticity, gene expression and severity of drought phenotypes of juvenile trees are likely not indicative of what occur in mature trees. Nevertheless, the results of this study improve our understandings on the molecular mechanisms underlying xylem plasticity in response to drought and rewatering and provide insights into the adaptations of other tree species to future drier climate.

## Data Availability

The datasets presented in this study can be found in online repositories. The names of the repository/repositories and accession number(s) can be found in the article/**Supplementary Material**.

## Supplementary material

Supplementary Figure 1 Concentrations of MDA **(A)**, O_2_^•-^ **(B)**, proline **(C)** and H_2_O_2_ **(D)** in the wood of drought-resistant Wubu and drought-susceptible Zhongshen1 exposed to 80, 50 and 15% field capacity for 21 days and re-watered to 80% field capacity (denoted as 80%r, 50%r and 15%r) for 12 days. The bar indicates mean ± SE (n = 6). Different letters on the bars indicate significant difference. ANOVAs of cultivar (C), soil water content (W), and their interaction (C × W) are also indicated. **P* < 0.05; ***P* < 0.01; ****P* < 0.001; *****P* < 0.0001; ns, not significant.

Supplementary Figure 2 Significantly enriched GO terms of the common DEGs in cambial zone of two mulberry cultivars under drought **(A)** and rewatering **(B)**.

Supplementary Figure 3 Key gene modules for drought recovery traits (xylem anatomy and the physiological responses of cambium and xylem) screened by WGCNA. Cluster dendrogram of expressed genes based on WGCNA analysis **(A)**, the number of genes in each module **(B)** and the correlation analysis between gene modules and traits **(C)** are shown. Pearson correlation coefficient (r) and *p* values (in brackets) are presented for each module. VF, vessel frequency; VLA, vessel lumen area; VLD, vessel lumen diameter; XT, xylem thickness; Starch-w, starch in wood; Starch-cam, starch in cambium.

Supplementary Figure 4 qRT-PCR analysis of 15 DEGs to validate RNA-seq results. The histogram and left axis are the relative expressions measured by qRT-PCR, and the dot-line and right axis are the FPKM value obtained by RNA-Seq. Error bars show the mean ± SD of three biological replicates. Different letters on the bars indicate significant differences. ANOVAs of cultivar (C), soil water content (W) and their interaction (C × W) are based on qRT-PCR data. **P* < 0.05; ***P* < 0.01; ****P* < 0.001; *****P* < 0.0001; ns, not significant. SPS1, SUCROSE-PHOSPHATE SYNTHASE 1; SPP2, SUCROSE-6-PHOSPHATASE 2; INV, ACID BETA-FRUCTOFURANOSIDASE; SS, SUCROSE SYNTHASE; AGPASE, GLUCOSE-1-PHOSPHATE ADENYLYL-TRANSFERASE SMALL SUBUNIT; ISO1, Isoamylase 1; BAM1, B-AMYLASE 1; HK, HEXOKINASE; FRK2, FRUCTOKINASE 2; AGAL, A-GALACTOSIDASE; BGAL9, B-GALACTOSIDASE 9; MAN, ENDO-1,4-B-MANNOSIDASE.
