# Supplementary figures and images for: Transcriptome-based WGCNA reveals the molecular regulation of xylem plasticity in acclimation to drought and rewatering in mulberry
Source: Front Plant Sci. 2024 Dec 18;15:1512645. doi: 10.3389/fpls.2024.1512645 (PMC11688357; doi:10.3389/fpls.2024.1512645)

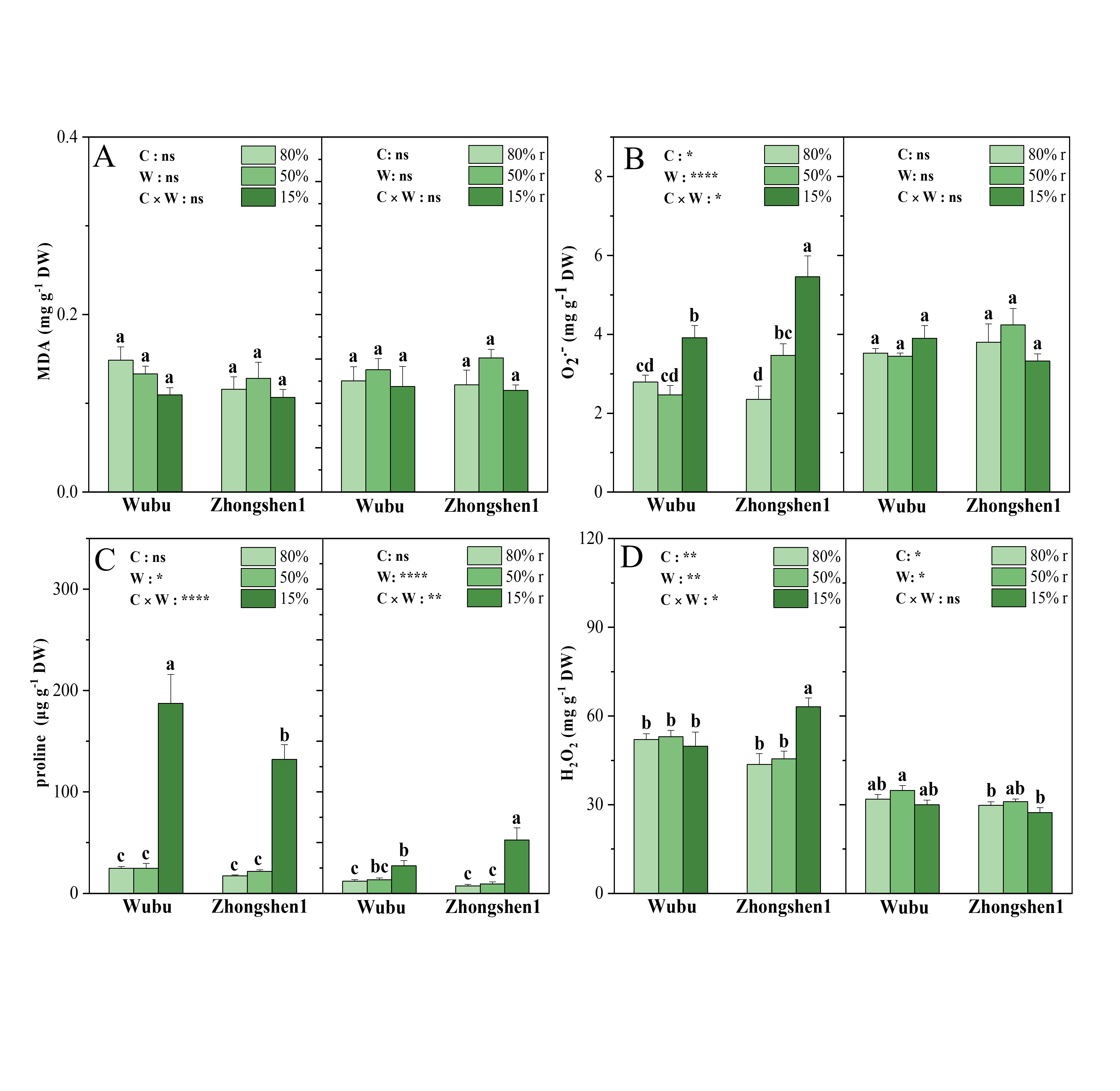

Supplement: Supplementary Figure 1 — Concentrations of MDA (A), O2 •- (B), proline (C) and H2O2 (D) in the wood of drought-resistant Wubu and drought-susceptible Zhongshen1 exposed to 80, 50 and 15% field capacity for 21 days and re-watered to 80% field capacity (denoted as 80%r, 50%r and 15%r) for 12 days. The bar indicates mean ± SE (n = 6). Different letters on the bars indicate significant difference. ANOVAs of cultivar (C), soil water content (W), and their interaction (C × W) are also indicated. *P < 0.05; **P < 0.01; ***P < 0.001; ****P < 0.0001; ns, not significant. [file Image1.tif]

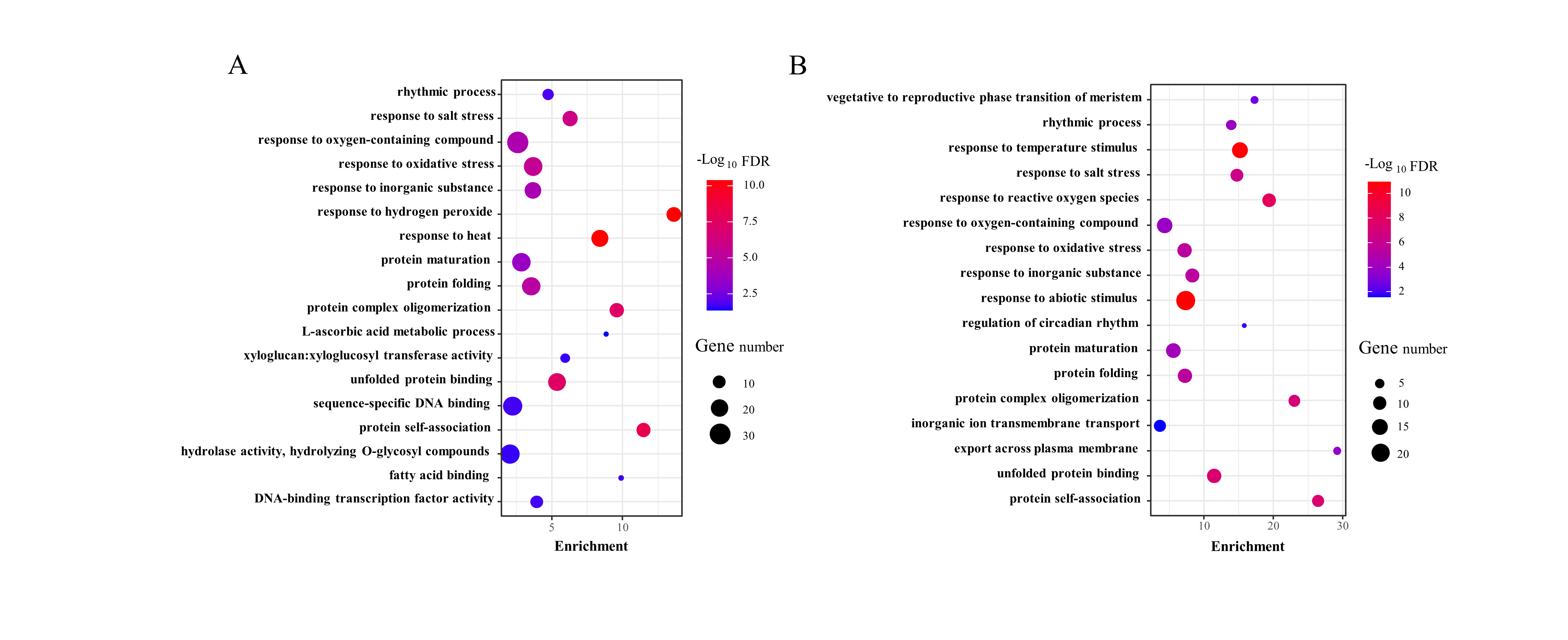

Supplement: Supplementary Figure 2 — Significantly enriched GO terms of the common DEGs in cambial zone of two mulberry cultivars under drought (A) and rewatering (B). [file Image2.tif]

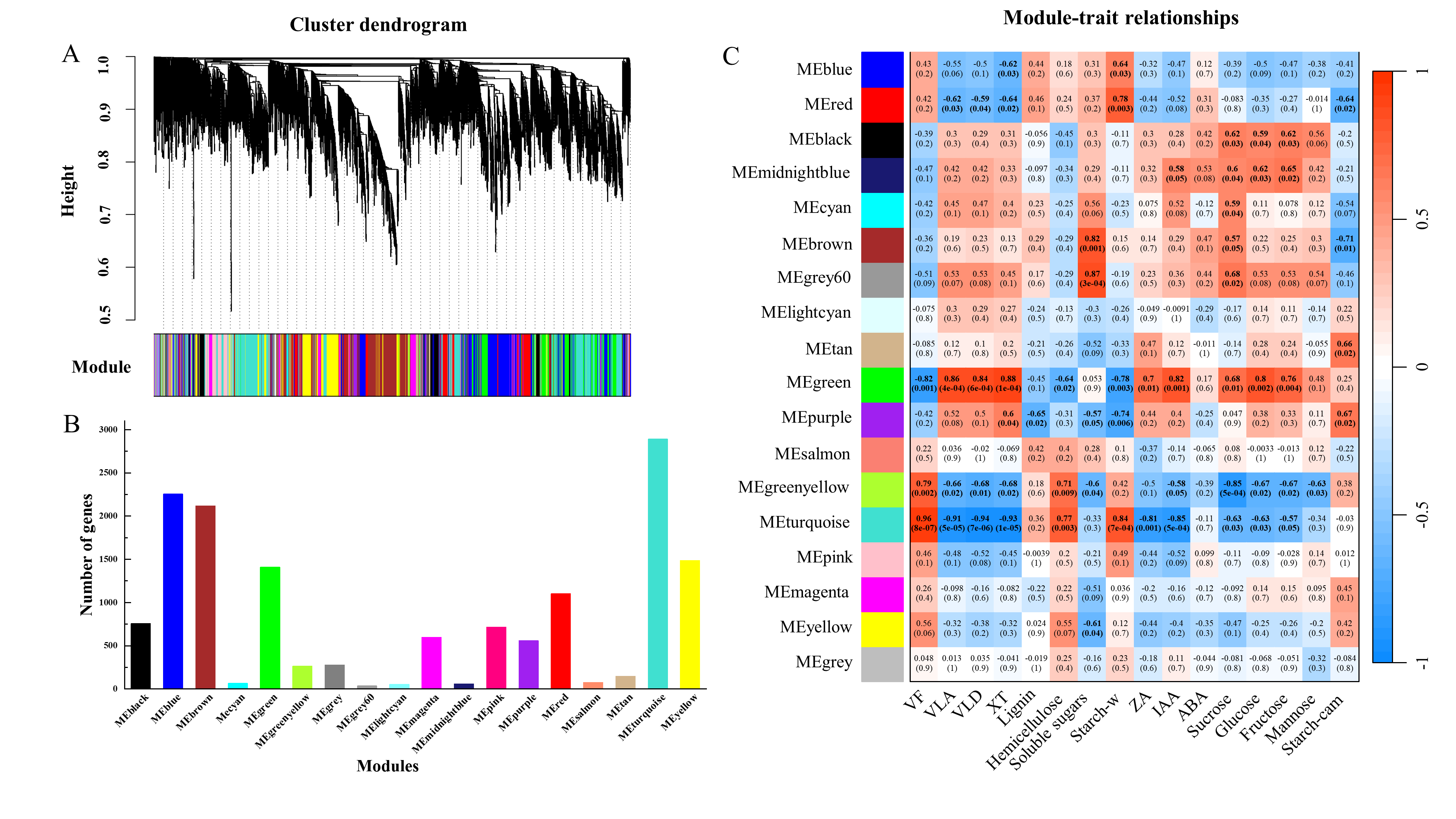

Supplement: Supplementary Figure 3 — Key gene modules for drought recovery traits (xylem anatomy and the physiological responses of cambium and xylem) screened by WGCNA. Cluster dendrogram of expressed genes based on WGCNA analysis (A), the number of genes in each module (B) and the correlation analysis between gene modules and traits (C) are shown. Pearson correlation coefficient (r) and p values (in brackets) are presented for each module. VF, vessel frequency; VLA, vessel lumen area; VLD, vessel lumen diameter; XT, xylem thickness; Starch-w, starch in wood; Starch-cam, starch in cambium. [file Image3.tif]

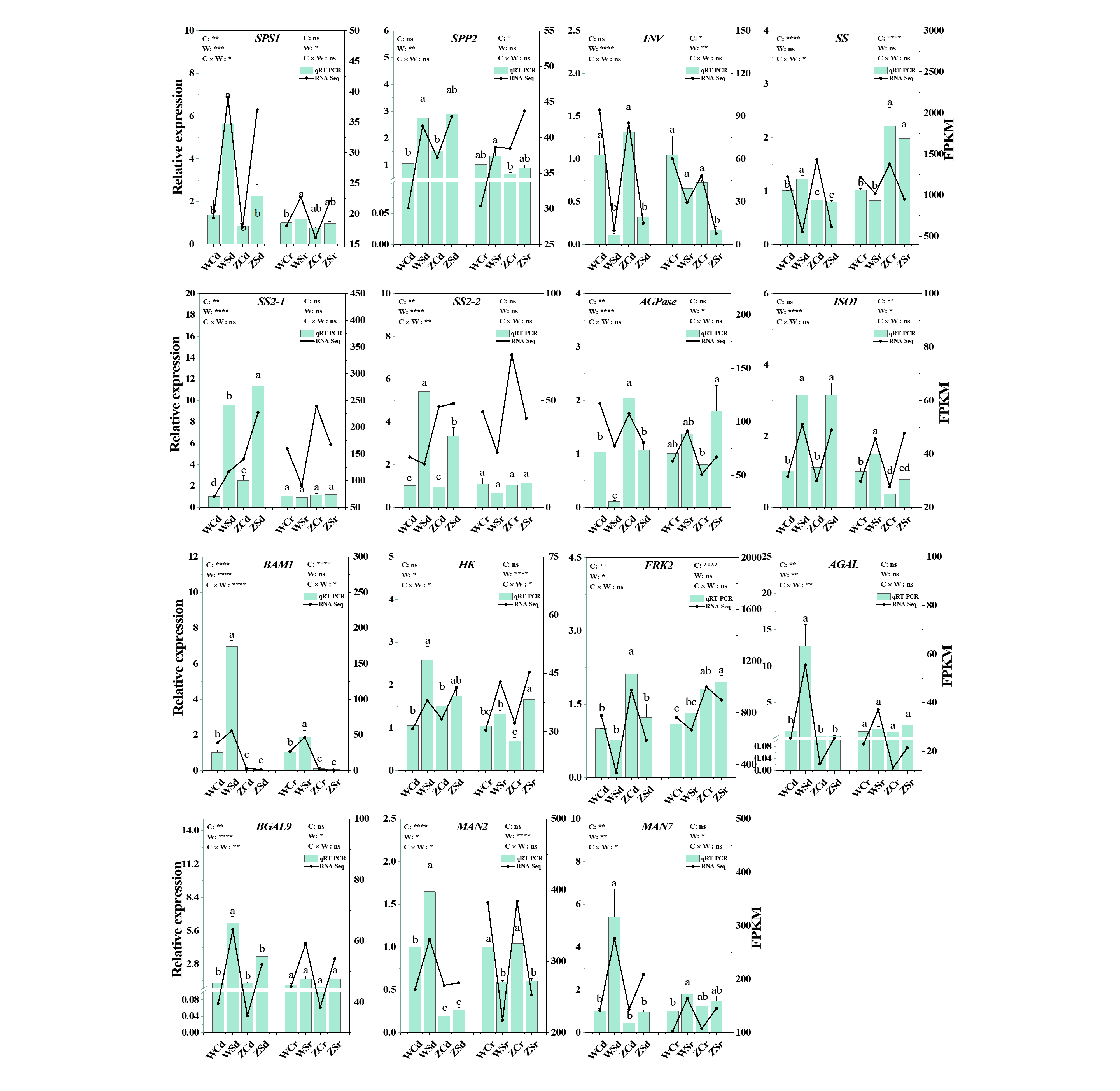

Supplement: Supplementary Figure 4 — qRT-PCR analysis of 15 DEGs to validate RNA-seq results. The histogram and left axis are the relative expressions measured by qRT-PCR, and the dot-line and right axis are the FPKM value obtained by RNA-Seq. Error bars show the mean ± SD of three biological replicates. Different letters on the bars indicate significant differences. ANOVAs of cultivar (C), soil water content (W) and their interaction (C × W) are based on qRT-PCR data. *P < 0.05; **P < 0.01; ***P < 0.001; ****P < 0.0001; ns, not significant. SPS1, SUCROSE-PHOSPHATE SYNTHASE 1; SPP2, SUCROSE-6-PHOSPHATASE 2; INV, ACID BETA-FRUCTOFURANOSIDASE; SS, SUCROSE SYNTHASE; AGPASE, GLUCOSE-1-PHOSPHATE ADENYLYL-TRANSFERASE SMALL SUBUNIT; ISO1, Isoamylase 1; BAM1, B-AMYLASE 1; HK, HEXOKINASE; FRK2, FRUCTOKINASE 2; AGAL, A-GALACTOSIDASE; BGAL9, B-GALACTOSIDASE 9; MAN, ENDO-1,4-B-MANNOSIDASE. [file Image4.tif]
